# Novel Lipid-Based Carriers of Provitamin D_3_: Synthesis and Spectroscopic Characterization of Acylglycerol Conjugated with 7-Dehydrocholesterol Residue and Its Glycerophospholipid Analogue

**DOI:** 10.3390/molecules29235805

**Published:** 2024-12-09

**Authors:** Witold Gładkowski, Susanna Ortlieb, Natalia Niezgoda, Anna Chojnacka, Paulina Fortuna, Paweł Wiercik

**Affiliations:** 1 Department of Food Chemistry and Biocatalysis, Wrocław University of Environmental and Life Sciences, Norwida 25, 50-375 Wrocław, Poland; natalia.niezgoda@upwr.edu.pl (N.N.); anna.chojnacka@upwr.edu.pl (A.C.); 2 Research Institute of Textile Chemistry and Textile Physics, University of Innsbruck, Hoechsterstraße 73, 6850 Dornbirn, Austria; susanna.ortlieb@uibk.ac.at; 3 Omics Research Center, Wrocław Medical University, 50-368 Wrocław, Poland; paulina.fortuna@umw.edu.pl; 4 Institute of Environmental Engineering, Wrocław University of Environmental and Life Sciences, Grunwaldzki Square 24, 50-363 Wrocław, Poland; pawel.wiercik@upwr.edu.pl

**Keywords:** lipid prodrugs, 7-dehydrocholesterol, modified acylglycerols, modified phospholipids, succinate linker, acyl migration

## Abstract

The aim of this research was to design and synthesize new lipid conjugates of 7-DHC that could serve as a new storage form of esterified provitamin D_3_, increasing the reservoir of this biomolecule in the epidermis and enabling controlled production of vitamin D_3_ even during periods of sunlight deficiency. Acylglycerol and glycerophospholipid containing succinate-linked provitamin D_3_ at the *sn*-2 position of the glycerol backbone were synthesized from dihydroxyacetone (DHA) and *sn*-glycerophosphocholine (GPC), respectively. The three-step synthesis of 1,3-dipalmitoyl-2-(7-dehydrocholesterylsuccinoyl)glycerol involved the esterification of DHA with palmitic acid, reduction of the carbonyl group, and conjugation of the resulting 1,3-dipalmitoylglycerol with 7-dehydrocholesterol hemisuccinate (7-DHC HS). The use of NaBH_3_CN as a reducing agent was crucial to avoid acyl migration and achieve the final product with 100% regioisomeric purity. For the preparation of 1-palmitoyl-2-(7-dehydrocholesterylsuccinoyl)-*sn*-glycero-3-phosphocholine, a two-step process was applied, involving the esterification of GPC at the *sn*-1 position with palmitic acid, followed by the conjugation of 1-palmitoyl-*sn*-glycero-3-phosphocholine with 7-DHC HS. Alongside the main product, a small amount of its regioisomer with provitamin D_3_ linked at the *sn*-1 position and palmitic acid at the *sn*-2 position was detected, indicating acyl migration from the *sn*-1 to the *sn*-2 position in the intermediate 1-palmitoyl*-sn*-glycerophosphocholine. The synthesized novel lipids were fully characterized using spectroscopic methods. They can find applications as novel lipid-based prodrugs as additives to sunscreen creams.

## 1. Introduction

Research highlights the importance of vitamin D not only in the prevention of rickets [1] and musculoskeletal disorders [2] but also in modulating immune responses [3,4] and participating in anticancer processes [5,6]. Its deficiency is associated with serious health consequences, including diabetes [7], cardiovascular diseases [8], autoimmune disorders [9] and cancers [10]. The primary source of vitamin D_3_ for humans is its production in the skin under ultraviolet radiation (UVB, 280–310 nm), which triggers the isomerization of provitamin D_3_ (7-dehydrocholesterol, 7-DHC) to previtamin D_3_, followed by thermal isomerization to vitamin D_3_ [11].

Vitamin D deficiency is becoming increasingly common due to low sun exposure during winter, and the use of sunscreen creams that protect the skin from UV radiation, which reduces endogenous production of this compound. For this reason, there is a growing interest in developing novel delivery systems for vitamin D, including oral administration, food fortification and transdermal delivery [12].

As demonstrated in investigations using an animal model, over 80% of provitamin D_3_ and vitamin D_3_ in the skin are present in the storage form of fatty acid esters. It was found that, similar to free provitamin D_3_, esters of provitamin D_3_ also undergo photoisomerization to produce vitamin D_3_ esters. These esters serve as a storage form of vitamin D_3_ ensuring a reservoir of free cholecalciferol during periods of low sunlight availability, as they are gradually hydrolyzed in the epidermis in the presence of the transport protein DBP. This protein binds the released vitamin D_3_ molecule in the blood serum and transports it to the liver, where it undergoes hydroxylation at the C-25 carbon atom [13]. The described mechanism of controlled release of free vitamin D_3_ slows its metabolism, limits its toxicity by regulating its concentration in the body, and, through its successive transformation into the active 25-hydroxy derivative, prevents vitamin D deficiency even during periods of limited sunlight.

Taking these findings into account, the aim of our research is to design and synthesize 7-DHC esterified with acylglycerols and phospholipids, which could serve as a new storage form of esterified provitamin D_3_ in the skin. Such lipid-based prodrugs of 7-DHC, prepared in the form of liposome formulations, can potentially be used as additives in sunscreen creams, increasing the amount of provitamin D_3_ in the epidermis and enabling controlled production of vitamin D_3_ even during periods of sunlight deficiency. In this paper, we present the synthesis and structural characterization of two 7-DHC derivatives: an acylglycerol-based prodrug in which 7-DHC is linked through the *sn*-2 position of the glycerol backbone and its glycerophospholipid-based analogue.

The production of symmetrical glyceride prodrugs is a common strategy for developing e triglyceride-based prodrugs. Following this approach, several acylglycerols have been synthesized with bioactive molecules conjugated at the *sn*-2 position, including, e.g., non-steroidal anti-inflammatory drugs (NSAIDs) [14,15,16], the anti-HIV agent didanosine [17], the antineoplastic agent chlorambucil [18], the antibacterial drug norfloxacin [19], and testosterone [20]. Similarly, the conjugation of bioactive molecules at the *sn*-2 position is employed to produce phospholipid-based prodrugs, e.g., containing NSAIDs like indomethacin [21], ibuprofen, or naproxen [22], anticonvulsant valproic acid [23], or the antibiotic fumagillin [24].

## 2. Results and Discussion

Our strategy for designing the target compounds involved the synthesis of 1,3-dipalmitoylglycerol and 1-palmitoyl-*sn*-glycero-3-phosphocholine followed by their conjugation with 7-dehydrocholesterol at the *sn*-2 position. We decided to attach 7-DHC residue via a succinyl linker, as this approach was successfully applied in our laboratory for the synthesis of acylglycerols containing stigmasterol [25,26,27]. The conjugation of bioactive molecules via succinyl linker to produce sterol-modified phospholipids has been previously reported for modified phospholipids containing residues of cholesterol [28], stigmasterol [29] or dehydroepiandrosterone (DHEA) [30].

### 2.1. Synthesis of 7-Dehydrocholesterol Hemisuccinate

7-Dehydrocholesterol hemisuccinate (7-DHC HS) was obtained in the reaction of 7-dehydrocholesterol with succinic anhydride in anhydrous pyridine in the presence of DMAP. The procedure generally followed the method described previously for stigmasterol hemisuccinate [25], but the purification step required modification. While crystallization from methanol afforded the product in only a 32% yield, flash chromatography was employed instead, significantly improving the yield to 76%. Tian et al. [31] reported the synthesis of 7-DHC HS but did not provide details of the synthetic procedure, and the ^1^H NMR description was incomplete. Therefore, in this paper, we present full spectroscopic data, including NMR, IR, and HRMS (Section 3.3.1 in Materials and Methods).

### 2.2. Synthesis of 1,3-Dipalmitoyl-2-(7-Dehydrocholestyrylsuccinoyl)glycerol

**Scheme 1:**
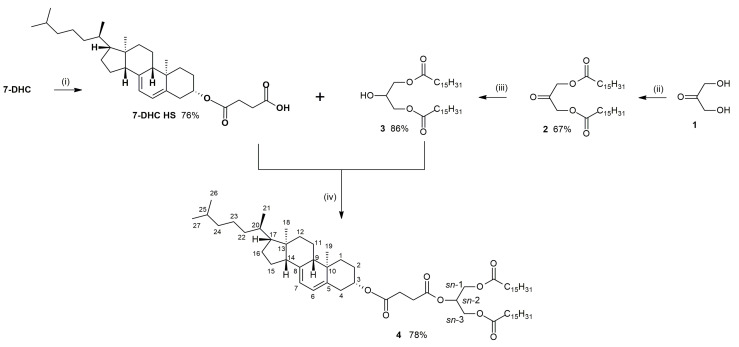
Synthesis of acylglycerol **4** containing succinyl-linked 7-DHC at the sn-2 position. Reagents and conditions: (i) succinic anhydride, DMAP, anhydrous pyridine, 60 °C, 24 h (ii) palmitic acid, DCC, DMAP, CHCl_3_, r.t., 24 h (iii) NaBH_3_CN, THF, glacial MeCOOH, r.t., 40 min. (iv) DCC, DMAP, CHCl_3_, r.t., 24 h.

In most synthetic pathways leading to modified symmetrical glycerides, including those developed by our research team for the synthesis of stigmasterol-modified acylglycerols [26,27], the starting material was dihydroxyacetone (DHA). Typically, DHA was esterified with a fatty acid, reduced to 1,3-diacylglycerol, and conjugated with the appropriate drug. A similar procedure was applied for the synthesis of the acylglycerol with the 7-DHC residue described here (Scheme 1).

Contrary to previous reports where DHA (**1**) was esterified with palmitoyl chloride in anhydrous pyridine [14,32], in our study, we used palmitic acid in the presence of DCC and DMAP to obtain the known 1,3-dipalmitoyloxypropan-2-one (**2**) [33] in a 67% yield after purification by flash chromatography. The Steglich procedure had been successfully applied earlier for the synthesis of 1,3-dimirystoyloxypropan-2-one [26] and 1,3-dioleoyloxypropan-2-one [27]. Detailed spectroscopic analysis (IR, NMR, HRMS) confirmed the structure of the obtained diester **2**. Upon reviewing the literature, we did not find any spectroscopic data of 1,3-dipalmitoyloxypropan-2-one (**2**) synthesized by other research groups; therefore, we decided to report them in this paper (Section 3.3.2 in Materials and Methods).

The next step involved the reduction of the carbonyl group of **2** to obtain 1,3-dipalmitoylglycerol. When sodium borohydride was used as the reducing agent, in addition to the desired 1,3-diacylglycerols, we observed the formation of their 1,2-regioisomers due to acyl group migration from the *sn*-1 to *sn*-2 position. A similar phenomenon was previously reported by Cockman et al. [34], as well as in our earlier investigations during the reduction of 1,3-diacyloxypropan-2-ones [26,27]. To avoid the necessity of regioisomeric diacylglycerol separation by flash chromatography and to improve the yield of the desired 1,3-isomer, we performed the reduction of **2** with sodium cyanoborohydride in tetrahydrofuran at pH 4, following the procedure proposed by Cockman et al. [34]. Both TLC analysis of the reaction mixture and NMR analysis showed no acyl migration, and 1,3-dipalmitoylglycerol was isolated as the only product in an 86% yield without requiring time-consuming chromatographic purification. The spectroscopic data of **2** were fully consistent with those published earlier [27].

In the final step, 1,3-dipalmitoylglycerol (**3**) was esterified with 7-DHC HS using the typical Steglich procedure. After flash chromatography, pure 1,3-dipalmitoyl-2-(7-dehydrocholesterylsuccinoyl)glycerol (**4**) was obtained in a 78% yield.

### 2.3. Synthesis of 1-Palmitoyl-2-(7-Dehydrocholesterylsuccinoyl)-sn-Glycero-3-Phosphocholine (***7***)

**Scheme 2:**
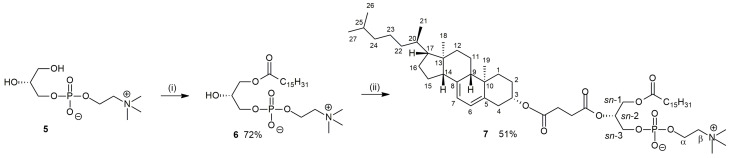
Synthesis of glycerophospholipid **7** containing succinyl-linked 7-DHC at the sn-2 position. Reagents and conditions: (i) DBTO, ^i^PrOH, reflux, 1 h; then TEA, palmitoyl chloride, r.t., 0.5 h; (ii) DCC, DMAP, CH_2_Cl_2_, 4 °C, 96 h.

A two-step process was employed to synthesize 1-palmitoyl-2-(7-dehydrocholesterylsuccinoyl)-*sn*-glycero-3-phosphocholine **7** (Scheme 2).

The first step involved the preparation of 1-palmitoyl-*sn*-glycero-3-phosphocholine (1-PA GPC) following the method previously described by Niezgoda [35]. In this process, *sn*-glycerophosphocholine (GPC) was initially transformed into a cyclic acetal intermediate and then selectively acylated with palmitoyl chloride in the presence of triethylamine (TEA). The resulting 1-PA GPC was immediately purified by flash chromatography to minimize acyl migration from the *sn*-1 to the *sn*-2 position. After removing water by evaporation with anhydrous ethanol, the final product was obtained in a 72% yield. The second step involved the Steglich esterification of 1-PA GPC with 7-DHC HS at 4 °C to form the final product. Despite using relatively high molar ratios of DCC and DMAP relative to 1-PA-GPC and a prolonged reaction time (48 h), TLC analysis indicated only partial conversion of the substrate. Adding one additional equivalent of DCC and extending the reaction time to 96 h resulted in the product being obtained in an unsatisfactory yield of 16%.

Carbodiimides, such as DCC, are prone to hydrolysis in the presence of water, which reduces the overall efficiency of the esterification [36]. Therefore, we concluded that the low yield might have been caused by insufficient drying of the substrates. To address this, in the next trial, 1-PA GPC was rigorously dried by adding different anhydrous solvents, including acetone, ethanol, and dichloromethane, and evaporating the azeotropes using a rotary evaporator. This procedure was repeated several times, followed by drying in a vacuum desiccator. The dried substrate was then directly subjected to Steglich esterification. Even after this procedure, relatively high molar ratios of DCC and DMAP and the long reaction time (96 h) were still required to drive the reaction to completion. This necessity is likely due to the reduced reactivity of the substrates at low temperatures, as the reaction was conducted at 4 °C to further limit acyl migration in 1-PA GPC. These improvements significantly increased the isolated yield of phospholipid **7** to 51%.

### 2.4. Spectroscopic Identification of the Final Products ***4*** and ***7***

HRMS analysis of both compounds confirmed their molecular masses. Detailed NMR measurements, including 2D spectroscopy (COSY, HMQC, and HMBC), allowed us to identify the signals corresponding to the glycerol backbone, the 7-DHC residue, palmitic acid, the succinyl linker, and, for the phospholipid **7**, the phosphocholine moiety of the molecule.

On ^1^H NMR spectra, the presence of the palmitic acid residue was confirmed by characteristic signals originating from one chain (phospholipid **7**) or two chains (acylglycerol **4**). Among these, particularly notable were two triplets: one from the terminal methyl group at 0.87 ppm and another from the methylene protons at the α carbons, appearing at 2.31 ppm (acylglycerol **4**) or 2.27 ppm (phospholipid **7**). On the spectra of both products, multiplets corresponding to the CH_2_ group at the β position were observed in the range of 1.54–1.60 ppm, while the signals from other CH_2_ protons of the acyl chains appeared as overlapping multiplets in the range of 1.18–1.31 ppm. On ^13^C NMR spectra, the most characteristic signals from the palmitic acid residue included those of the carbonyl carbons from the ester group at 173.3 ppm (acylglycerol **4**) or 173.6 ppm (phospholipid **7**), the α and β carbons (34.0 and 24.8 ppm for acylglycerol **4,** and 34.0 and 24.9 ppm for phospholipid **7**), and the ω-2, ω-1, and ω carbons, respectively, for both acylglycerol **4** and phospholipid **7** (31.9, 22.7, and 14.1 ppm).

As the chemical shifts of signals from the sterol part of acylglycerol **4** observed on ^13^C NMR spectra were fully consistent with those reported by Wilson et al. for 7-dehydrocholesterol acetate [37], we could unequivocally assign them to the corresponding carbons. Consequently, using data from HMQC and HMBC spectra, we also identified the signals of specific protons on the ^1^H NMR spectra. Among these, the most distinctive was a triplet of triplets from H-3 at 4.71 ppm, along with signals from olefinic protons: a doublet of triplets at 5.38 ppm from H-7 and a doublet of doublets at 5.56 ppm from H-6. Separate signals were also observed for both protons of the CH_2_-4 group (2.36 and 2.49 ppm), H-12β (2.08 ppm), H-9 (1.98 ppm), H-25 (1.52 ppm), and H-22 (1.01 ppm). Additionally, singlets from methyl groups at C-18 and C-19 (0.61 and 0.94 ppm, respectively) were found, as well as doublets from methyl groups at C-26 and C-27 (0.86 and 0.87 ppm) and C-21 (0.93 ppm). The signals from the remaining protons of the sterol part were superimposed, but through 2D spectroscopy analysis, their locations within specific multiplets were determined and found to be consistent with the data reported by Wilson et al. for 7-dehydrocholesterol acetate [37]. A similar assignment was made for the carbons and protons of the sterol residue in the spectra of phospholipid **7**.

For both synthesized lipids, signals from the succinyl linker were observed, including multiplets of two methylene groups in the range of 2.55–2.66 ppm in the ^1^H NMR spectra and signals of carbonyl carbons in the ^13^C NMR spectra, at 171.4 and 171.5 ppm for acylglycerol **4,** as well as 171.6 and 171.9 ppm for phospholipid **7**. The HMBC spectrum of compound **4** showed correlations between the peaks at 171.4 and 171.5 ppm and the signals of H-3 and H_sn-2_, confirming the attachment of the 7-DHC hemisuccinate to the *sn*-2 position of the glycerol backbone. Analogous HMBC correlations were observed for compound **7**.

The presence of the phosphoric acid residue in phospholipid **7** was confirmed by the signal at −0.95 ppm in the ^31^P NMR spectrum, and the choline residue was identified by a singlet from three methyl groups attached to the nitrogen atom at 3.33 ppm, along with two broad singlets from methylene groups CH_2_-α and CH_2_-β at 4.28 ppm and 3.76 ppm, respectively, in the ^1^H NMR spectrum. In addition to the singlet at 54.33 ppm from the carbons of the three methyl groups, the ^13^C spectroscopy also showed signals from the corresponding carbons of the choline fragment, represented by characteristic doublets with ^13^C-^31^P coupling constants ^2^*J* = 3.3 Hz for the C-α atom and ^3^*J* = 6.1 Hz for the C-β atom.

**Figure 1:**
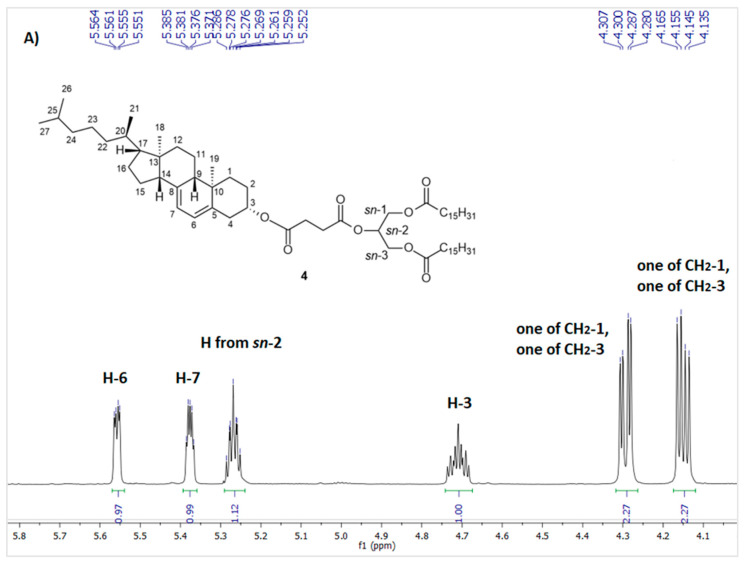

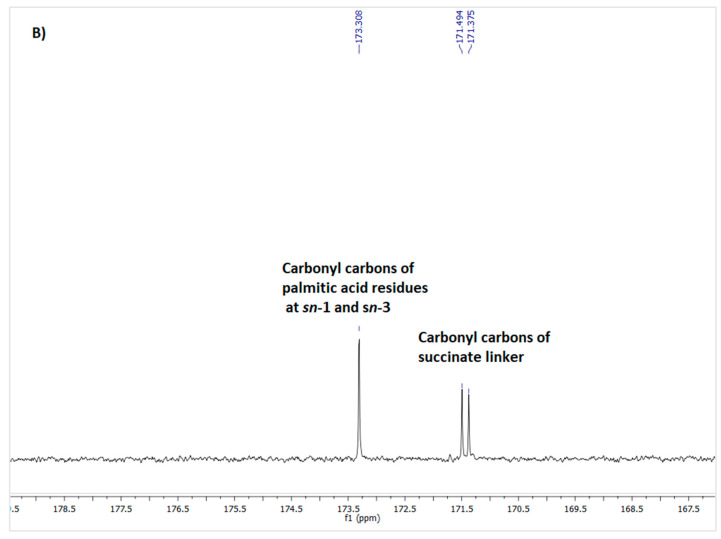
Fragment of ^1^H NMR (**A**) and ^13^C NMR (**B**) spectrum of acylglycerol **4**.

Valuable information confirming the symmetrical structure of **4** was provided by the analysis of signals from the atoms of the glycerol backbone. A non-stereoselective reduction of the dipalmitoyloxypropan-2-one (**2**), followed by Steglich esterification, generated a prochiral center at C-2. Consequently, in the ^1^H NMR spectrum of **4,** two doublets of doublets at 4.15 and 4.25 ppm (Figure 1A) were attributed to the diastereotopic pairs of protons at the *sn*-1 and *sn*-3 positions. In the ^13^C NMR spectra (Figure 1B), only one signal from the carbons at the *sn*-1 and *sn*-3 positions was observed (61.9 ppm). Similarly, the carbonyl carbons of the palmitic acyl residues at sn-1 and sn-3 were also represented by a single signal at 173.3 ppm. These data fully confirmed the presence of the 7-DHC residue at the sn-2 position and the absence of any acyl migration during the reaction. Similar observations were reported in our previous paper to confirm the symmetrical structure of 1,3-diacyl-2-stigmasterylsuccinoylglycerols [26,27].

**Figure 2:**
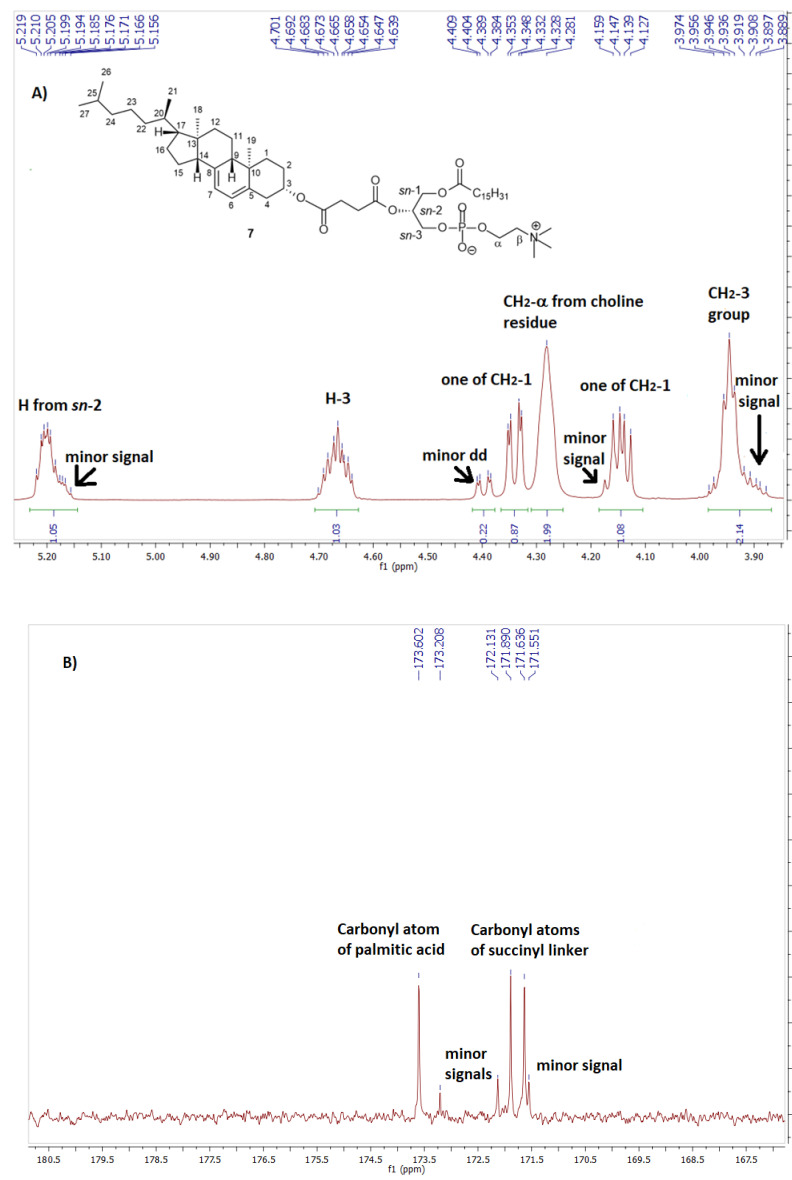
Fragment of ^1^H NMR (**A**) and ^13^C NMR (**B**) spectrum of phospholipid **7**.

In the case of phospholipid **7**, the analysis of the spectral range characteristic of protons in the glycerol skeleton as well as of the carbonyl carbons of the ester moieties was highly informative. In the ^1^H NMR spectra of phospholipid **7** (Figure 2A), the protons of the CH_2_ group at the *sn*-3 position resonated in the range of 3.92–3.98 ppm, whereas those of the CH_2_ group at the *sn*-1 position were observed as two distinct doublets of doublets at 4.14 ppm and 4.34 ppm. The multiplet corresponding to the *sn*-2 proton was significantly shifted downfield to 5.20 ppm. The carbon-phosphorus coupling produced characteristic doublets in the ^13^C NMR spectrum: for C*_sn_*_-2_ at 71.2 ppm (^3^*J* = 7.4 Hz) and for C*_sn_*_-3_ at 63.5 ppm (^2^*J* = 4.0 Hz).

Careful spectral analysis of phospholipid **7** allowed us to identify several minor signals in the resonance region of the glycerol protons (Figure 2A). The most distinct and visible was a doublet of doublets at 4.40 ppm, shifted downfield relative to the major signal from one of the CH_2_-1 protons by 0.06 ppm. In contrast, a minor signal at 5.17 ppm was shifted slightly upfield by 0.03 ppm compared to the major signal from H-2. Other minor signals were more or less overlapped with their neighboring major signals. Similarly, in the ^13^C NMR spectrum, apart from the major signals of the carbonyl carbons, three minor signals were also observed in this region (Figure 2B).

The above-mentioned minor signals in the NMR spectra led us to the assumption that they originated from the corresponding protons of regioisomeric phospholipid, containing the 7-DHC residue attached to the *sn*-1 position and palmitic acid linked to the *sn*-2 position. To support our hypothesis, we compared the chemical shifts of the minor and major signals detected in the spectra of phospholipid **7** with the chemical shifts of corresponding signals reported for analogs containing dehydroepiandrosterone (DHEA). (Table 1). We considered two regiosomeric glycerophospholipids: one containing DHEA at the *sn*-2 position and oleic acid at the *sn*-1 position (1-OA-2-DHEA PChol) and the other with DHEA at the *sn*-1 and oleic acid at the *sn*-2 position (1-DHEA-2-OA PChol), synthesized by Kłobucki et al. [30].

Comparing the spectra of the glycerophospholipid containing a fatty acid residue at the *sn*-2 position and sterol molecule at the *sn*-1 position (1-DHEA-2-OL-PChol) to its regioisomer, one of CH_2_-1 protons resonates at a lower field (4.39 ppm versus 4.36 ppm), while proton H-2 resonates at a higher field (5.18 ppm versus 5.20 ppm). On the other hand, in the spectra of the regioisomer with the sterol residue at the *sn*-2 position (1-OA-2-DHEA-PChol), compared to 1-sterol glycerophospholipid (1-DHEA-2-OL-PChol), the difference in chemical shifts between signals from carbonyl carbons of the succinate linker is significantly smaller (Δδ = 0.1 versus 0.4), and the signal from the carbon atom of the fatty acid residue is significantly shifted upfield (173.6 versus 173.2). Similar differences in chemical shifts between the minor and major signals observed in the analyzed spectra of phospholipid **7** confirmed that, in addition to the expected product (major signals), a small amount of 2-palmitoyl-1-(7-dehydrocholesterylsuccinoyl)-*sn*-glycero-3-phosphocholine (minor signals) was formed.

The formation of this regioisomer can be explained by the partial transformation of the intermediate 1-palmitoyl-*sn*-glycero-3-phosphocholine (1-PA GPC) (**6**) into 2-palmitoyl-*sn*-glycero-3-phosphocholine (2-PA GPC) through the acyl migration of the palmitic acid residue, followed by the subsequent Steglich esterification of 2-PA GPC. The confirmation of this rearrangement is evident in the NMR spectra of **6**, which show minor signals from the glycerol backbone in the ^1^H NMR spectrum, a minor signal from the ester carbon of the palmitic acid moiety at *sn*-2 in the ^13^C NMR spectrum, and a minor signal of phosphorous from orthophosphate unit in the ^31^P NMR (Supporting Information, Figures S19–S21). These signals are also present in the spectra of **6** reported by Papangelis et al., who observed partial migration between the *sn*-1 and *sn*-2 positions of 1-PA GPC [38].

Acyl migration in lysophospholipids between the *sn*-1 and *sn*-2 positions is a common phenomenon observed during chemical and enzymatic modifications of phospholipids [35,39,40,41,42] leading to a regioisomeric mixtures of products and reducing the yield and purity of the final glycerophospholipids. The equilibrium between 1-acyl GPC and 2-acyl GPC typically stabilizes at a ratio of approximately 9:1 [43]. This isomerization is favored by factors such as alkaline or acidic pH, temperature, and solvent polarity [44]. Kiełbowicz et al. [45] observed acyl migration in lysophospholipids containing palmitic acid in various organic solvents, including isopropanol and dichloromethane, which were also used in the synthesis described here. Additionally, the migration can be promoted by silica gel used during chromatographic purification [38]. Sugasini and Subbaiah studied the effect of the acyl group on migration and found that saturated fatty acids, such as palmitic acid, are more susceptible to migration than polyunsaturated fatty acids [46].

### 2.5. State of the Art and Future Perspectives on Synthesized Lipid Carriers of Provitamin D_3_

Previous studies have focused on the use of vitamin D_3_ or provitamin D_3_ as carriers for anticancer drugs. Nanocarriers based on conjugates of vitamin D_3_ with doxorubicin or paclitaxel have been developed as platforms for drug delivery in cancer therapy [47]. In liposomal systems, 7-DHC can replace cholesterol, enhancing the anticancer activity of photosensitizer-encapsulated liposomes through a combination of photodynamic therapy and photoactivated chemotherapy [48].

The novel acylglycerols and glycerophospholipids obtained in this research can serve as new storage forms of esterified provitamin D_3_ for delivery to the skin. However, we plan to conduct comprehensive in vitro and in vivo toxicity studies in future research to ensure the safety of these lipids for dermatological and pharmaceutical applications. To develop transdermal formulations, efficient delivery systems will be designed. Due to their structure, these lipids also serve as an excellent base for use as components of liposomes or nanosomes, which may enhance the stability and efficiency of delivering 7-DHC to the human body, improving skin penetration and controlling the release of active compounds. The obtained nanocarriers will be evaluated for stability, size, and thermotropic parameters, with additional bioassays to be performed. The use of synthesized lipids in sunscreen formulations supporting vitamin D synthesis in the skin will also be assessed. Furthermore, tests will be conducted to evaluate the efficacy of controlled release of provitamin D_3_.

In future work, we plan to optimize the composition of lipid-based carriers of provitamin D_3_, including structural modifications of acyl chains or changing the length of the linker to enhance the chemical and biological stability of the synthesized lipid carriers.

## 3. Materials and Methods

### 3.1. Chemicals

Dihydroxyacetone (≥98%), palmitic acid (≥99%), 7-dehydrocholesterol (≥95%), 4-(dimethylamino)pyridine (DMAP, ≥99%), *N*,*N*′-dicyclohexylcarbodiimide (DCC, 99%), sodium cyanoborohydride, dibutyltin(IV) oxide (DBTO, 98%), triethylamine (TEA, ≥99,5%), palmitoyl chloride (98%), DOWEX^®^ 50WX8 hydrogen form, ethanol-free chloroform (≥99%), anhydrous tetrahydrofuran (THF, ≥99.9%), anhydrous dichloromethane (DCM, ≥99.8%), anhydrous pyridine (99.8%), and hexane (ACS reagent, ≥99%) were purchased from Merck (Darmstadt, Germany). *sn*-Glycero-3-phosphocholine (GPC) was purchased from Bachem AG (Bubendorf, Switzerland). Acetic acid (99.5–99.9%) and isopropanol were purchased from POCH (Gliwice, Poland). Anhydrous isopropanol was prepared by dissolving sodium (8 g/L) in alcohol previously dried by distillation from CaSO_4_. The solution was then distilled again, and the fresh distillate was stored with MS 3A molecular sieves beads for 96 h to obtain isopropanol containing only 9 ppm of water. Other chemicals of analytical grade were purchased from Chempur (Piekary Śląskie, Poland).

### 3.2. Analytical Methods

The progress of the reactions was reviewed by Thin Layer Chromatography (TLC) using 0.2 mm aluminum plates coated with silica gel 60 F_254_ (Merck, Darmstadt, Germany). Chromatograms were visualized by spraying the plates with a solution of 1% Ce(SO_4_)_2_ and 2% H_3_[P(Mo_3_O_10_)_4_] in 10% H_2_SO_4_, followed by heating the plates to 120–200 °C or with a 0.05% solution of primuline in an acetone:water mixture (4:1, *v*/*v*), making the spots visible under UV light (λ = 365 nm).

Reaction products were purified by flash chromatography using the puriFlash^®^ SX520 Plus Interchim system (Interchim, Montluçon, France), equipped with a gradient pump, UV detector, and fraction collector. Samples were dry-loaded on a pre-column (puriFlash^®^ F0012), and products were separated on puriFlash^®^ SIHP 30 μm columns using gradient elution with solvent mixtures as described in the respective method sections (flow rate: 26 mL/min; pressure: 15 mbar). Nuclear Magnetic Resonance spectra (^1^H NMR, ^13^C NMR, COSY, HMQC, HMBC) were recorded on a Bruker Avance II 600 MHz spectrometer (Bruker, Rheinstetten, Germany) or Jeol 400 MHz Year Hold Magnet spectrometer (Jeol Ltd., Tokyo, Japan). Chemical shifts were referenced to the residual solvent signal (CDCl_3_, δ_H_ = 7.26, δ_C_ = 77.00). Infrared spectroscopy (IR) was performed on Nicolet iS10 FTIR Spectrometer (Thermo Scientific™, Waltham, MA, USA) equipped with a monolithic diamond ATR crystal attachment. High Resolution Mass Spectra (HRMS) were recorded on Bruker Daltonics ESI-Q-TOF maXis impact mass spectrometer (Bruker, Billerica, MA, USA) or Waters Xevo G2 mass spectrometer (Waters, Milford, MA, USA) using positive electrospray ionization (ESI) techniques.

### 3.3. Experimental Procedures

#### 3.3.1. Synthesis of 7-Dehydrocholesteryl Hemisuccinate (7-DHC HS)

7-Dehydrocholesterol (1.5 g, 3.9 mmol), succinic anhydride (1.37 g, 13.7 mmol), and DMAP (0.45 g, 3.9 mmol) were dissolved in 40 mL of anhydrous pyridine. The reaction was carried out using a magnetic stirrer (Heidolph, Schwabach, Germany) at 60 °C in a thermostated glycerol bath under a reflux condenser for 24 h. Once the substrate had reacted completely (TLC, chloroform/methanol/acetic acid 95:5:0.1), the reaction mixture was acidified with 1 M HCl to pH 2. The product was extracted with DCM, and the organic layer was washed with brine until neutral and dried over anhydrous MgSO_4_. After filtration and solvent evaporation under vacuum, the crude product was purified by flash chromatography using a gradient elution system (from hexane:acetone:AcOH 100:0:0 to hexane:acetone:AcOH, 4:1:0.01, *v*/*v*/*v*), yielding pure 7-dehydrocholesteryl hemisuccinate (1.14 g, yield 76%) with the following physical and spectroscopic data:

White crystals, mp 155–160 °C, *R_f_* = 0.18 (hexane:acetone:AcOH, 4:1:0.01); ^1^H NMR (600 MHz, CDCl_3_) δ: 0.61 (s, 3H, CH_3_-18), 0.86 and 0.87 (two d, *J* = 6.6 Hz, 6H, CH_3_-26 and CH_3_-27), 0.93 (d, *J* = 6.7 Hz, 3H, CH_3_-21), 0.94 (s, 3H, CH_3_-19), 1.02 (m, 1H, one of CH_2_-22), 1.07-1.17 (m, 3H, CH_2_-24 and one of CH_2_-23), 1.18–1.27 (m, 2H, H-17 and H-12α), 1.28–1.42 (m, 6H, H-16β, one of CH_2_-23, one of CH_2_-22, H-1α, H-15β and H-20), 1.52 (m, 1H, H-25), 1.55–1.61 (m, 2H, H-2β and H-11α), 1.66–1.74 (m, 2H, H-15α and H-11β), 1.85–1.99 (m, 4H, H-14α, H-1β, H-2α and H-16α), 1.98 (m, 1H, H-9), 2.08 (m, 1H, H-12β), 2.36 (m, 1H, H-4β), 2.49 (ddd, *J* = 14.5, 4.8 and 2.2 Hz, 1H, H-4α), 2.61 and 2.68 (two m, 4H, –O(O)C–CH_2_–CH_2_–COOH), 4.72 (m, 1H, H-3), 5.38 (dt, *J* = 5.6 and 2.5 Hz, 1H, H-7), 5.56 (dd, *J* = 5.6 and 2.2 Hz, 1H, H-6); ^13^C NMR (150 MHz, CDCl_3_) δ: 11.8 (C-18), 16.2 (C-19) 18.8 (C-21), 21.0 (C-11), 22.5 and 22.8 (C-26, C-27), 23.0 (C-15), 23.9 (C-23), 28.0 (C-25), 28.1 (C-2, C-16), 29.0 and 29.2 (–O(O)C–CH_2_–CH_2_–COOH), 36.1 (C-22), 36.1 (C-20), 36.5 (C-4), 37.0 (C-10), 37.9 (C-1), 39.1 (C-12), 39.5 (C-24), 42.9 (C-13), 46.0 (C-9), 54.4 (C-14), 55.9 (C-17), 73.3 (C-3), 116.3 (C-7), 120.3 (C-6), 138.3 (C-5), 141.6 (C-8), 171.6 (–O(O)C–CH_2_–CH_2_–COOH), 177.9 (–O(O)C–CH_2_–CH_2_–COOH); IR (ATR): ν_max_ = 2953, 1732, 1709, 1174, 1466 cm^−1^; HRMS (ESI): *m*/*z* calcd for C_31_H_48_O_4_: 507.3445 [M+Na]^+^; found: 507.3447.

#### 3.3.2. Synthesis of Target Acylglycerol with 7-Dehydrocholesterol Residue

##### Preparation of 1,3-Dipalmitoyloxypropan-2-one (**2**)

Dihydroxyacetone (0.5 g, 5.6 mmol), *N*,*N*′-dicyclohexylcarbodiimide (DCC, 2.66 g, 12.88 mmol), and 4-(dimethylamino)pyridine (DMAP, 1.57 g, 12.88 mmol) were dissolved in ethanol-free chloroform. Palmitic acid (3.35 g, 13.07 mmol) in ethanol-free chloroform was added drop-wise to the reaction mixture until the total volume of 100 mL. After 24 h of stirring at room temperature, complete substrate conversion was observed (TLC: hexane/ethyl acetate, 5:1; visualization by primuline test). The white precipitate was removed by filtration through a funnel with a G4 sintered disc. The filtrate was then washed with 0.5 M HCl, followed by saturated NaCl solution until neutral, dried with anhydrous MgSO_4_, filtered, and concentrated in vacuo. The purified product (2.13 g, 67% yield) was obtained by flash chromatography using a gradient elution method (from hexane:DCM, 100:0 to hexane:DCM, 35:65). Its physical and spectroscopic data are given below:

White crystals, mp 73–78 °C, lit. 77–78 °C [33], *R_f_* = 0.45 (hexane:DCM, 1:2), ^1^H NMR (400 MHz, CDCl_3_) δ: 0.88 (t, *J* = 6.9 Hz, 6H, 2 × -OC(O)(CH_2_)_14_CH_3_), 1.20–1.38 (m, 48H, 2 × -OC(O)CH_2_CH_2_(CH_2_)_12_CH_3_), 1.61–1.70 (m, 4H, 2 × -OC(O)CH_2_CH_2_(CH_2_)_12_CH_3_), 2.42 (t, *J* = 7.5 Hz, 4H, 2 × -OC(O)CH_2_(CH_2_)_13_CH_3_), 4.74 (s, 4H, CH_2_-1 and CH_2_-3); ^13^C NMR (100 MHz, CDCl_3_) δ: 14.1 (2 × -OC(O)(CH_2_)_14_CH_3_), 22.7 (2 × -OC(O)(CH_2_)_13_CH_2_CH_3_), 24.8 (2 × -OC(O)CH_2_CH_2_(CH_2_)_12_CH_3_), 29.0, 29.2, 29.3, 29.4, 29.6, 29.6, 29.7 (2 × -OC(O)CH_2_CH_2_(CH_2_)_10_CH_2_CH_2_CH_3_), 31.9 (2 × -OC(O)(CH_2_)_10_CH_2_CH_2_CH_3_), 33.7 (2 × -OC(O)CH_2_(CH_2_)_11_CH_3_), 66.1 (C-1, C-3), 172.9 (2 × -OC(O)(CH_2_)_14_CH_3_), 198.1 (C-2); IR (ATR): ν_max_ = 2916, 2850, 1746, 1732, 1709, 1175, 719 cm^−1^; HRMS (ESI): *m*/*z* calcd for C_35_H_66_O_5_: 567.4988 [M+H]^+^; found: 567.4982.

##### Preparation of 1,3-Dipalmitoylglycerol (**3**)

NaBH_3_CN (0.083 g, 1.3 mmol) was added portion-wise to the stirring solution of 1,3-dipalmitoyloxypropan-2-one (2) (0.5 g, 0.88 mmol) in THF (40 mL). Subsequently, a few mL of glacial acetic acid were added until the reaction mixture was acidified to pH 4. After 40 min, when no substrate was observed by TLC (hexane:ethyl acetate, 5:1; primuline), the solvent was evaporated in vacuo. The residue was suspended in water, and the product was extracted by DCM. After the work-up of the organic layer (washing with brine, drying with anhydrous MgSO_4_, and solvent evaporation), 0.43 g (86% yield) of pure 1,3-dipalmitoylglycerol was obtained.

Physical data: white crystals, mp 66–69 °C, lit. 68–70 °C [27], *R_f_* = 0.3 (hexane:ethyl acetate, 5:1), spectroscopic data were consistent with those reported in our previous paper [27].

##### Preparation of 1,3-Dipalmitoyl-2-(7-Dehydrocholesterylsuccinoyl)glycerol (**4**)

7-DHC HS (0.192 g, 0.40 mmol) dissolved in ethanol-free chloroform was added in portions to the stirring mixture of 1,3-dipalmitoylglycerol (3) (0.15 g, 0.26 mmol), DCC (0.083 g, 0.40 mmol) and DMAP (0.053 g, 0.43 mmol) in the same solvent (total volume 20 mL). The solution was stirred at room temperature for 24 h, and after complete substrate consumption (TLC: hexane:ethyl acetate, 5:1), the reaction was worked up as described for 1,3-dipalmitoylpropanone. Pure product (0.211 g, yield 78%) was obtained by flash chromatography using a gradient elution system (from hexane:DCM, 100:0 to hexane:DCM, 20:80). Its physical and spectroscopic data are as follows:

Waxy solid, *R_f_* = 0.55 (hexane:ethyl acetate, 5:1); ^1^H NMR (600 MHz, CDCl_3_) δ: 0.61 (s, 3H, CH_3_-18), 0.86 and 0.87 (two d, *J* = 6.6 Hz, 6H, CH_3_-26 and CH_3_-27), 0.87 (t, *J* = 6.9 Hz, 6H, 2 × -OC(O)(CH_2_)_14_CH_3_), 0.93 (d, *J* = 6.9 Hz, 3H, CH_3_-21), 0.94 (s, 3H, CH_3_-19), 1.00 (m, 1H, one of CH_2_-22), 1.06–1.17 (m, 3H, CH_2_-24 and one of CH_2_-23), 1.18–1.31 (m, 50H, H-17 and H-12α and 2 × -OC(O)CH_2_CH_2_(CH_2_)_12_CH_3_), 1.31–1.42 (m, 6H, H-16β, one of CH_2_-23, one of CH_2_-22, H-1α, H-15β and H-20), 1.52 (m, 1H, H-25), 1.55–1.64 (m, 6H, H-2β, H-11α and 2 × -OC(O)CH_2_CH_2_(CH_2_)_12_CH_3_), 1.66–1.74 (m, 2H, H-15α and H-11β), 1.85–1.95 (m, 4H, H-14α, H-1β, H-2α and H-16α), 1.98 (m, 1H, H-9), 2.08 (ddd, *J* = 12.5, 4.3 and 2.5 Hz, 1H, H-12β), 2.31 (t, *J* = 7.6 Hz, 4H, 2 × -OC(O)CH_2_(CH_2_)_13_CH_3_), 2.36 (m, 1H, H-4β), 2.49 (ddd, *J* = 14.5, 4.5 and 2.3 Hz, 1H, H-4α), 2.58–2.66 (m, 4H, -OC(O)CH_2_CH_2_(O)CO-DChol), 4.15 (dd, *J* = 11.9 and 5.9 Hz, 2H, one of CH_2_ at *sn*-1 and one of CH_2_ at *sn*-3), 4.29 (dd, *J* = 11.9 and 3.8 Hz, 2H, one of CH_2_ at *sn*-1 and one of CH_2_ at *sn*-3), 4.71 (tt, *J* = 11.5 and 4.5 Hz, 1H, H-3), 5.27 (tt, *J* = 5.9 and 3.8 Hz, 1H, H*_sn_*_-2_), 5.38 (dt, *J* = 5.6 and 2.6 Hz, 1H, H-7), 5.56 (dd, *J* = 5.6 and 2.3 Hz, 1H, H-6); ^13^C NMR (150 MHz, CDCl_3_) δ: 11.8 (C-18), 14.1 (2 × -OC(O)(CH_2_)_14_CH_3_), 16.1 (C-19) 18.8 (C-21), 21.0 (C-11), 22.5 and 22.8 (C-26, C-27), 22.7 (2 × -OC(O)(CH_2_)_13_CH_2_CH_3_), 23.0 (C-15), 23.9 (C-23), 24.8 (2 × -OC(O)CH_2_CH_2_(CH_2_)_12_CH_3_), 28.0 (C-25), 28.1 (C-2, C-16), 29.1, 29.1, 29.3, 29.4, 29.5, 29.6, 29.7 and 29.7 (-OC(O)CH_2_CH_2_(O)CO-DChol, 2 × -OC(O)CH_2_CH_2_(CH_2_)_10_CH_2_CH_2_CH_3_), 31.9 (2 × -OC(O)(CH_2_)_12_CH_2_CH_2_CH_3_), 34.0 (2 × -OC(O)CH_2_(CH_2_)_13_CH_3_), 36.1 (C-22), 36.1 (C-20), 36.6 (C-4), 37.0 (C-10), 37.9 (C-1), 39.1 (C-12), 39.5 (C-24), 42.9 (C-13), 46.0 (C-9), 54.4 (C-14), 55.9 (C-17), 61.9 (C*_sn_*_-1_ and C*_sn_*_-3_), 69.5 (C*_sn_*_-2_), 73.2 (C-3), 116.3 (C-7), 120.3 (C-6), 138.3 (C-5), 141.6 (C-8), 171.4 and 171.5 (-OC(O)CH_2_CH_2_(O)CO-DChol), 173.3 (2 × -OC(O)(CH_2_)_14_CH_3_); IR (ATR): ν_max_ = 2919, 2850, 1735, 1468, 1155 cm^−1^; HRMS (ESI): *m*/*z* calcd for C_66_H_114_O_8_: 1057.8406 [M+Na]^+^; found: 1057.8398

#### 3.3.3. Synthesis of Glycerophospholipid with 7-Dehydrocholesterol Residue

##### Preparation of 1-Palmitoyl-*sn*-Glycero-3-Phosphocholine (**6**)

The synthesis of 1-palmitoyl-*sn*-glycero-3-phosphocholine was carried out according to the procedure described by Niezgoda et al. [35]. *sn*-Glycero-3-phosphocholine (**5**, 0.95 g, 3.7 mmol) was dried by repeated (3x) evaporation with an anhydrous DCM and then suspended with DBTO (0.92 g, 3.7 mmol) in anhydrous isopropanol (15 mL) and refluxed for 1 h. The reaction mixture was cooled to room temperature and TEA (5.5 mmol) was added followed by palmitoyl chloride (1.52 g, 5.5 mmol). After 0.5 h of the reaction, the organic solvent was evaporated from the reaction mixture under vacuum. The crude residue was purified by flash chromatography using a gradient elution system (from DCM:MeOH:H_2_O, 95:5:0 to DCM:MeOH:H_2_O, 65:30:5) and dried by the evaporation from anhydrous solvents (acetone, EtOH, DCM) followed by overnight drying in a vacuum desiccator to obtain 1-palmitoyl-2-hydroxy-*sn*-glycero-3-phosphocholine (1.29 g, yield 72%).

Physical data: white solid, *R_f_* = 0.22 (DCM:MeOH:H_2_O, 65:30:5), spectroscopic data were consistent with literature values [38].

##### Preparation of 1-Palmitoyl-2-(7-Dehydrocholesterylsuccinoyl)-*sn*-Glycero-3-Phosphocholine (**7**)

To the stirring mixture of rigorously dried 1-palmitoyl-2-hydroxy-*sn*-glycero-3-phosphocholine (**6**, 0.15 g, 0.3 mmol), 7-DHC HS (0.308 g, 0.64 mmol), and DMAP (0.08 g, 0.65 mmol) dissolved in 9 mL of anhydrous DCM, a solution of DCC (0.131 g, 0.63 mmol) in 3 mL of DCM was added. The reaction was carried out for 96 h at 4 °C. The resulting precipitate was filtered off using a Shott funnel with a G4 sintered disc, and Dowex^®^ 50WX8 H^+^ resin was added to the reaction mixture. After 30 min of stirring, the resin was filtered off, and the solvent was evaporated under vacuum. The crude product was purified by flash chromatography using a gradient elution system (from DCM:MeOH:H_2_O, 95:5:0 to DCM:MeOH:H_2_O, 65:30:5), yielding pure 1-palmitoyl-2-(7-dehydrocholesteryl)succinoyl-*sn*-glycero-3-phosphocholine (0.147 g, yield 51%) with following physical and spectroscopic data:

Waxy white solid, *R_f_* = 0.51 (DCM:MeOH:H_2_O, 65:30:5); ^1^H NMR (600 MHz, CDCl_3_) δ: 0.61 (s, 3H, CH_3_-18), 0.85 and 0.86 (two d, *J* = 6.6 Hz, 6H, CH_3_-26 and CH_3_-27), 0.87 (t, *J* = 7.0 Hz, 3H, -OC(O)(CH_2_)_14_CH_3_), 0.93 (d, *J* = 6.3 Hz, 3H, CH_3_-21), 0.94 (s, 3H, CH_3_-19), 1.01 (m, 1H, one of CH_2_-22), 1.06–1.17 (m, 3H, CH_2_-24 and one of CH_2_-23), 1.18–1.31 (m, 26H, H-17 and H-12α and -OC(O)CH_2_CH_2_(CH_2_)_12_CH_3_), 1.31–1.41 (m, 6H, H-16β, one of CH_2_-23, one of CH_2_-22, H-1α, H-15β and H-20), 1.51 (m, 1H, H-25), 1.54–1.60 (m, 4H, H-2β, H-11α and -OC(O)CH_2_CH_2_(CH_2_)_12_CH_3_), 1.64–1.74 (m, 2H, H-15α and H-11β), 1.84–1.93 (m, 4H, H-14α, H-1β, H-2α and H-16α), 1.97 (m, 1H, H-9), 2.08 (m, 1H, H-12β), 2.27 (t, *J* = 7.5 Hz, 2H, -OC(O)CH_2_(CH_2_)_13_CH_3_), 2.35 (m, 1H, H-4β), 2.47 (m, 1H, H-4α), 2.55–2.66 (m, 4H, -OC(O)CH_2_CH_2_(O)CO-DChol), 3.33 (s, 9H, -N(CH_3_)_3_), 3.76 (broad s, 2H, CH_2_-β), 3.92–3.98 (m, 2H, CH_2_ at *sn*-3), 4.14 (dd, *J* = 12.0 and 7.2 Hz, 1H, one of CH_2_ at *sn*-1), 4.28 (broad s, 2H, CH_2_-α), 4.34 (dd, *J* = 12.0 and 2.9 Hz, 1H, one of CH_2_ at *sn*-1), 4.67 (tt, *J* = 11.2 and 4.4 Hz, 1H, H-3), 5.20 (m, 1H, H*_sn_*_-2_), 5.37 (m, 1H, H-7), 5.55 (m, 1H, H-6); ^13^C NMR (150 MHz, CDCl_3_) δ: 11.8 (C-18), 14.1 (-OC(O)(CH_2_)_14_CH_3_), 16.1 (C-19), 18.8 (C-21), 21.0 (C-11), 22.5 and 22.8 (C-26, C-27), 22.7 (-OC(O)(CH_2_)_13_CH_2_CH_3_), 23.0 (C-15), 23.9 (C-23), 24.9 (-OC(O)CH_2_CH_2_(CH_2_)_12_CH_3_), 28.0 (C-25), 28.01 (C-2, C-16), 29.1, 29.2, 29.3, 29.4, 29.6, 29.7, 29.8 (-OC(O)CH_2_CH_2_(O)CO-DChol, -OC(O)CH_2_CH_2_(CH_2_)_10_CH_2_CH_2_CH_3_), 31.9 (-OC(O)(CH_2_)_12_CH_2_CH_2_CH_3_), 34.0 (-OC(O)CH_2_(CH_2_)_13_CH_3_), 36.1 (C-22), 36.2 (C-20), 36.6 (C-4), 37.0 (C-10), 37.9 (C-1), 39.1 (C-12), 39.5 (C-24), 42.9 (C-13), 46.0 (C-9), 54.3 (-N(**C**H_3_)_3_, 54.4 (C-14), 55.9 (C-17), 59.3 (d, *J* = 3.3 Hz, C-α), 62.7 (C*_sn_*_-1_), 63.5 (d, *J* = 4.0 Hz, C*_sn_*_-3_), 66.3 (d, *J* = 6.1 Hz, C-β), 71.2 (d, *J* = 7.4 Hz, C*_sn_*_-2_), 73.2 (C-3), 116.3 (C-7), 120.3 (C-6), 138.3 (C-5), 141.6 (C-8), 171.6 (-OC(O)CH_2_CH_2_(O)CO-DChol), 171.9 (-OC(O)CH_2_CH_2_(O)CO-DChol), 173.6 (-OC(O)(CH_2_)_14_CH_3_); ^31^P NMR (243 MHz, CDCl_3_) δ: −0.95; IR (ATR): ν_max_ = 3388, 2923, 2851, 1733, 1467, 1247, 1161, 1092 cm^−1^; HRMS (ESI): *m*/*z* calcd for C_55_H_96_NO_10_P: 962.6850 [M+H]^+^; found: 962.6898.

## 4. Conclusions

In conclusion, two novel lipid derivatives of provitamin D_3_ were successfully synthesized and characterized by spectroscopic analysis. The three-step synthesis of an acylglycerol containing two palmitic acid residues at the *sn*-1 and *sn*-3 positions and succinate-linked DHC at the *sn*-2 position proved to be efficient. Using NaBH_3_CN as the reducing agent prevented acyl migration in 1,3-dipalmitoylglycerol and resulted in the exclusive formation of the desired regioisomer, acylglycerol **4**. For the preparation of a glycerophospholipid with DHC linked to the *sn*-2 position (**7**), different techniques should be considered to prevent acyl migration. These include controlling the pH, using stabilizing agents such as borate, improving chromatographic conditions by employing modified stationary phases like a calcium-modified silica gel, or changing the solvent used for the synthesis of intermediate 1-PA GPC and its subsequent Steglich esterification.

## Figures and Tables

**Table 1:** Chemical shifts of selected major and minor signals observed in the spectra of phospholipid **7** and two analogous glycerophospholipids containing hemisuccinate-DHEA and an oleic acid residues (1-OA-2-DHEA-PChol ^1^ and 1-DHEA-2-OL-PChol ^2^).

Proton or Carbon	Chemical Shift [ppm]			
	Major Signals in the Spectrum of Phospholipid 7	1-OA-2-DHEA-PChol ^3^	Minor Signals in the Spectrum of Phospholipid 7	1-DHEA-2-OA-PChol ^3^
one of the proton from CH_2_ at the *sn*-1	4.14	4.14	Overlapped with 4.14	4.14
one of the proton from CH_2_ at the *sn*-1	4.34	4.36	4.40	4.39
H at the *sn*-2	5.20	5.20	5.17	5.18
carbonyl carbons from the hemisuccinate linker	171.6 and 171.9	171.6 and 171.7	171.5 and 172.1	171.5 and 171.9
carbonyl carbon from the fatty acid residue	173.6	173.6	173.2	173.2

^1^ 1-Oleoyl-2-(3β-O-succinylandrost-5-en-17-one)-*sn*-glycero-3-phosphocholine. ^2^ 1-(3β-O-Succinylandrost-5-en-17-one)-2-oleoyl-*sn*-glycero-3-phosphocholine. ^3^ Data from Kłobucki et al. [30].

## Data Availability

Data are contained within the article and Supplementary Materials.

## Supplementary Materials

The following supporting information can be downloaded at: https://www.mdpi.com/article/10.3390/molecules29235805/s1. Figure S1: ^1^H NMR spectrum of 1,3-dipalmitoyloxypropan-2-one (**2**); Figure S2: ^13^C NMR spectrum of 1,3-dipalmitoyloxypropan-2-one (**2**); Figure S3: DEPT 135 spectrum of 1,3-dipalmitoyloxypropan-2-one (**2**); Figure S4: COSY spectrum of 1,3-dipalmitoyloxypropan-2-one (**2**); Figure S5: HMQC spectrum of 1,3-dipalmitoyloxypropan-2-one (**2**); Figure S6: HMBC spectrum of 1,3-dipalmitoyloxypropan-2-one (**2**); Figure S7: ^1^H NMR spectrum of 7-dehydrocholesterol hemisuccinate (**7-DHC HS**); Figure S8: ^13^C NMR spectrum of 7-dehydrocholesterol hemisuccinate (**7-DHC HS**); Figure S9: DEPT 135 NMR spectrum of 7-dehydrocholesterol hemisuccinate (**7-DHC HS**); Figure S10: COSY spectrum of 7-dehydrocholesterol hemisuccinate (**7-DHC HS**); Figure S11: HMQC spectrum of 7-dehydrocholesterol hemisuccinate (**7-DHC HS**); Figure S12: HMBC spectrum of 7-dehydrocholesterol hemisuccinate (**7-DHC HS**); Figure S13: ^1^H NMR spectrum of 1,3-dipalmitoyl-2-(7-dehydrocholestyrylsuccinoyl)glycerol (**4**); Figure S14: ^13^C NMR spectrum of 1,3-dipalmitoyl-2-(7-dehydrocholestyrylsuccinoyl)glycerol (**4**); Figure S15: DEPT 135 spectrum of 1,3-dipalmitoyl-2-(7-dehydrocholestyrylsuccinoyl)glycerol (**4**); Figure S16: COSY spectrum of 1,3-dipalmitoyl-2-(7-dehydrocholestyrylsuccinoyl)glycerol (**4**); Figure S17: HMQC spectrum of 1,3-dipalmitoyl-2-(7-dehydrocholestyrylsuccinoyl)glycerol (**4**); Figure S18: HMBC spectrum of 1,3-dipalmitoyl-2-(7-dehydrocholestyrylsuccinoyl)glycerol (**4**); Figure S19: ^1^H NMR spectrum of 1-palmitoyl-*sn*-glycero-3-phosphocholine (**6**); Figure S20: ^13^C NMR spectrum of 1-palmitoyl-*sn*-glycero-3-phosphocholine (**6**); Figure S21: ^31^P NMR spectrum of 1-palmitoyl-*sn*-glycero-3-phosphocholine (**6**); Figure S22: ^1^H NMR spectrum of 1-palmitoyl-2-(7-dehydrocholesterylsuccinoyl)-*sn*-glycero-3-phosphocholine (**7**); Figure S23: ^13^C NMR spectrum of 1-palmitoyl-2-(7-dehydrocholesterylsuccinoyl)-*sn*-glycero-3-phosphocholine (**7**); Figure S24: DEPT 135 spectrum of 1-palmitoyl-2-(7-dehydrocholesterylsuccinoyl)-*sn*-glycero-3-phosphocholine (**7**); Figure S25: COSY spectrum of 1-palmitoyl-2-(7-dehydrocholesterylsuccinoyl)-*sn*-glycero-3-phosphocholine (**7**); Figure S26: HMQC spectrum of 1-palmitoyl-2-(7-dehydrocholesterylsuccinoyl)-*sn*-glycero-3-phosphocholine (**7**); Figure S27: HMBC spectrum of 1-palmitoyl-2-(7-dehydrocholesterylsuccinoyl)-*sn*-glycero-3-phosphocholine (**7**); Figure S28: ^31^P NMR spectrum of 1-palmitoyl-2-(7-dehydrocholesterylsuccinoyl)-*sn*-glycero-3-phosphocholine (**7**); Figure S29: IR spectrum of 1,3-dipalmitoyloxypropan-2-one (**2**); Figure S30: IR spectrum of 7-dehydrocholesterol hemisuccinate (**7-DHC HS**); Figure S31: IR spectrum of 1,3-dipalmitoyl-2-(7-dehydrocholestyrylsuccinoyl)glycerol (**4**); Figure S32: IR spectrum of 1-palmitoyl-2-(7-dehydrocholesterylsuccinoyl)-*sn*-glycero-3-phosphocholine (**7**).
