# Supplementary material for: Novel Lipid-Based Carriers of Provitamin D3: Synthesis and Spectroscopic Characterization of Acylglycerol Conjugated with 7-Dehydrocholesterol Residue and Its Glycerophospholipid Analogue
Source: Molecules. 2024 Dec 9;29(23):5805. doi: 10.3390/molecules29235805 (PMC11643614; doi:10.3390/molecules29235805)
Supplement: Supplementary file 1 [file molecules-29-05805-s001.zip › molecules-3318735-supplementary.pdf]

Supporting Information

Novel Lipid-Based Carriers of Provitamin D_3: Synthesis and Spectroscopic Characterization of Acylglycerol Conjugated with 7-Dehydrocholesterol Residue and its Glycerophospholipid Analogue

Witold Gładkowski [1,*], Susanna Ortlieb [2], Natalia Niezgoda [1], Anna Chojnacka [1], Paulina Fortuna [3] and Paweł Wiercik [4]

[1] Department of Food Chemistry and Biocatalysis, Wrocław University of Environmental and Life Sciences, Norwida 25, 50-375 Wrocław, Poland; natalia.niezgoda@upwr.edu.pl (N.N.); anna.chojnacka@upwr.edu.pl (A.C.)
[2] Research Institute of Textile Chemistry and Textile Physics, University of Innsbruck, Hoechsterstraße 73, 6850 Dornbirn, Austria; susanna.ortlieb@uibk.ac.at
[3] Omics Research Center, Wrocław Medical University, 50-368 Wrocław, Poland; paulina.fortuna@umw.edu.pl
[4] Institute of Environmental Engineering, Wrocław University of Environmental and Life Sciences, Grunwaldzki Square 24, 50-363 Wrocław, Poland; pawel.wiercik@upwr.edu.pl
* Correspondence: witold.gladkowski@upwr.edu.pl; Tel.: +48-71-3205-154

Table of content

	Pages
^{1}H NMR spectrum of 1,3-dipalmitoyloxypropan-2-one (**2**) (Fig. S1)	**4**
^{13}C NMR spectrum of 1,3-dipalmitoyloxypropan-2-one (**2**) (Fig. S2)	**4**
DEPT 135 spectrum of 1,3-dipalmitoyloxypropan-2-one (**2**) (Fig. S3)	**5**
COSY spectrum of 1,3-dipalmitoyloxypropan-2-one (**2**) (Fig. S4)	**5**
HMQC spectrum of 1,3-dipalmitoyloxypropan-2-one (**2**) (Fig. S5)	**6**
HMBC spectrum of 1,3-dipalmitoyloxypropan-2-one (**2**) (Fig. S6)	**6**
^{1}H NMR spectrum of 7-dehydrocholesterol hemisuccinate (**7-DHC HS**) (Fig. S7)	**7**
^{13}C NMR spectrum of 7-dehydrocholesterol hemisuccinate (**7-DHC HS**) (Fig. S8)	**7**
DEPT 135 spectrum of 7-dehydrocholesterol hemisuccinate (**7-DHC HS**) (Fig. S9)	**8**
COSY spectrum of 7-dehydrocholesterol hemisuccinate (**7-DHC HS**) (Fig. S10)	**8**
HMQC spectrum of 7-dehydrocholesterol hemisuccinate (**7-DHC HS**) (Fig. S11)	**9**
HMBC spectrum of 7-dehydrocholesterol hemisuccinate (**7-DHC HS**) (Fig. S12)	**9**
^{1}H NMR spectrum of 1,3-dipalmitoyl-2-(7-dehydrocholestyrylsuccinoyl)glycerol (**4**) (Fig. S13)	**10**
^{13}C NMR spectrum of 1,3-dipalmitoyl-2-(7-dehydrocholestyrylsuccinoyl)glycerol (**4**) (Fig. S14)	**10**
DEPT 135 spectrum of 1,3-dipalmitoyl-2-(7-dehydrocholestyrylsuccinoyl)glycerol (**4**) (Fig. S15)	**11**
COSY spectrum of 1,3-dipalmitoyl-2-(7-dehydrocholestyrylsuccinoyl)glycerol (**4**) (Fig. S16)	**11**
HMQC spectrum of 1,3-dipalmitoyl-2-(7-dehydrocholestyrylsuccinoyl)glycerol (**4**) (Fig. S17)	**12**
HMBC spectrum of 1,3-dipalmitoyl-2-(7-dehydrocholestyrylsuccinoyl)glycerol (**4**) (Fig. S18)	**12**
^{1}H NMR spectrum of 1-palmitoyl-*sn*-glycero-3-phosphocholine (**6**) (Fig. S19)	**13**
^{13}C NMR spectrum of 1-palmitoyl-*sn*-glycero-3-phosphocholine (**6**) (Fig. S20)	**13**
^{31}P NMR spectrum of 1-palmitoyl-*sn*-glycero-3-phosphocholine (**6**) (Fig. S21)	**14**
^{1}H NMR spectrum of 1-palmitoyl-2-(7-dehydrocholesterylsuccinoyl)-*sn*-glycero-3-phosphocholine (**7**) (Fig. S22)	**14**
^{13}C NMR spectrum of 1-palmitoyl-2-(7-dehydrocholesterylsuccinoyl)-*sn*-glycero-3-phosphocholine (**7**) (Fig. S23)	**15**
DEPT 135 spectrum of1-palmitoyl-2-(7-dehydrocholesterylsuccinoyl)-*sn*-glycero-3-phosphocholine (**7**)) (Fig. S24)	**15**

COSY spectrum of 1-palmitoyl-2-(7-dehydrocholesterylsuccinoyl)-*sn*-glycero-3-phosphocholine (**7**) (Fig. S25)	**16**
HMQC spectrum of 1-palmitoyl-2-(7-dehydrocholesterylsuccinoyl)-*sn*-glycero-3-phosphocholine (**7**) (Fig. S26)	**16**
HMBC spectrum of 1-palmitoyl-2-(7-dehydrocholesterylsuccinoyl)-*sn*-glycero-3-phosphocholine (**7**) (Fig. S27)	**17**
^{31}P NMR spectrum of 1-palmitoyl-2-(7-dehydrocholesterylsuccinoyl)-*sn*-glycero-3-phosphocholine (**7**) (Fig. S28)	**17**
IR spectrum of 1,3-dipalmitoyloxypropan-2-one (**2**) (Fig. S29)	**18**
IR spectrum of 7-dehydrocholesterol hemisuccinate (**7-DHC HS**) (Fig. S30)	**18**
IR spectrum of 1,3-dipalmitoyl-2-(7-dehydrocholestyrylsuccinoyl)glycerol (**4**) (Fig. S31)	**19**
IR spectrum of 1-palmitoyl-2-(7-dehydrocholesterylsuccinoyl)-*sn*-glycero-3-phosphocholine (**7**) (Fig. S32)	**19**

NMR SPECTRA:

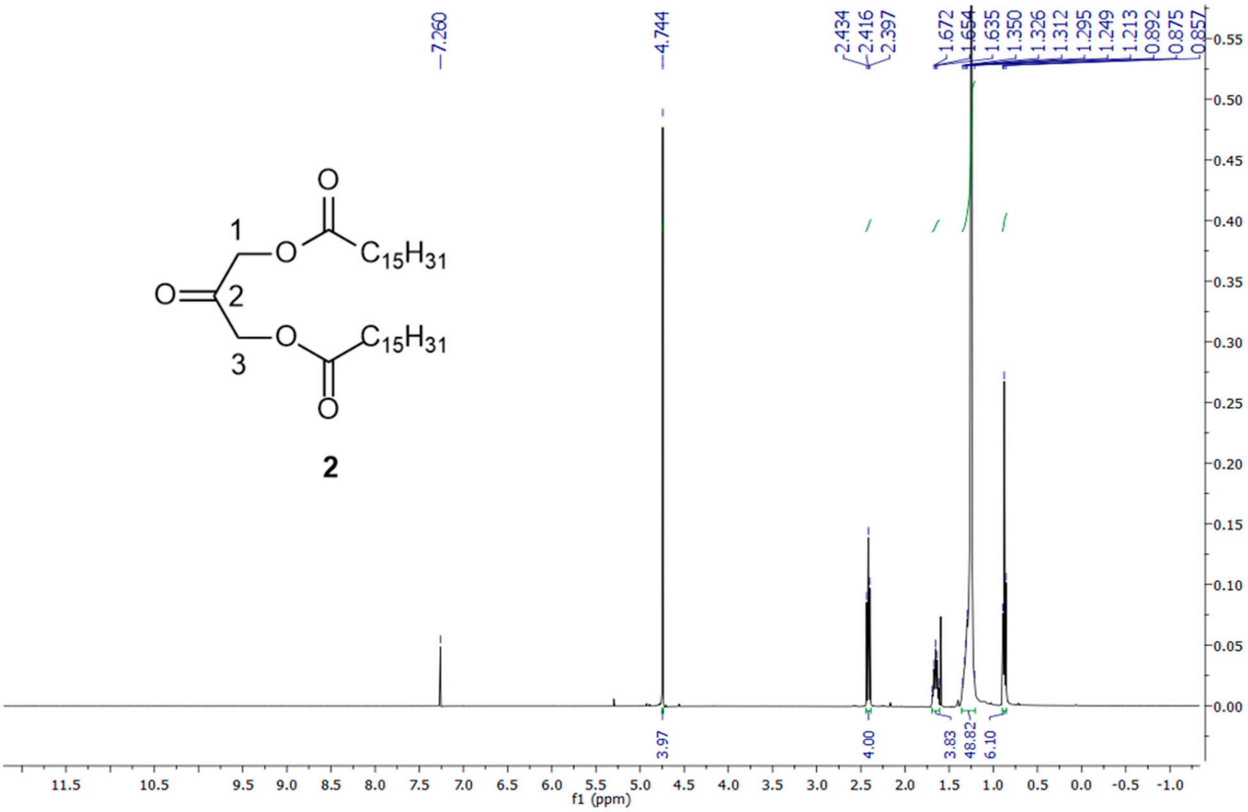

Figure S1. ^{1}H NMR spectrum of 1,3-dipalmitoyloxypropan-2-one (**2**)

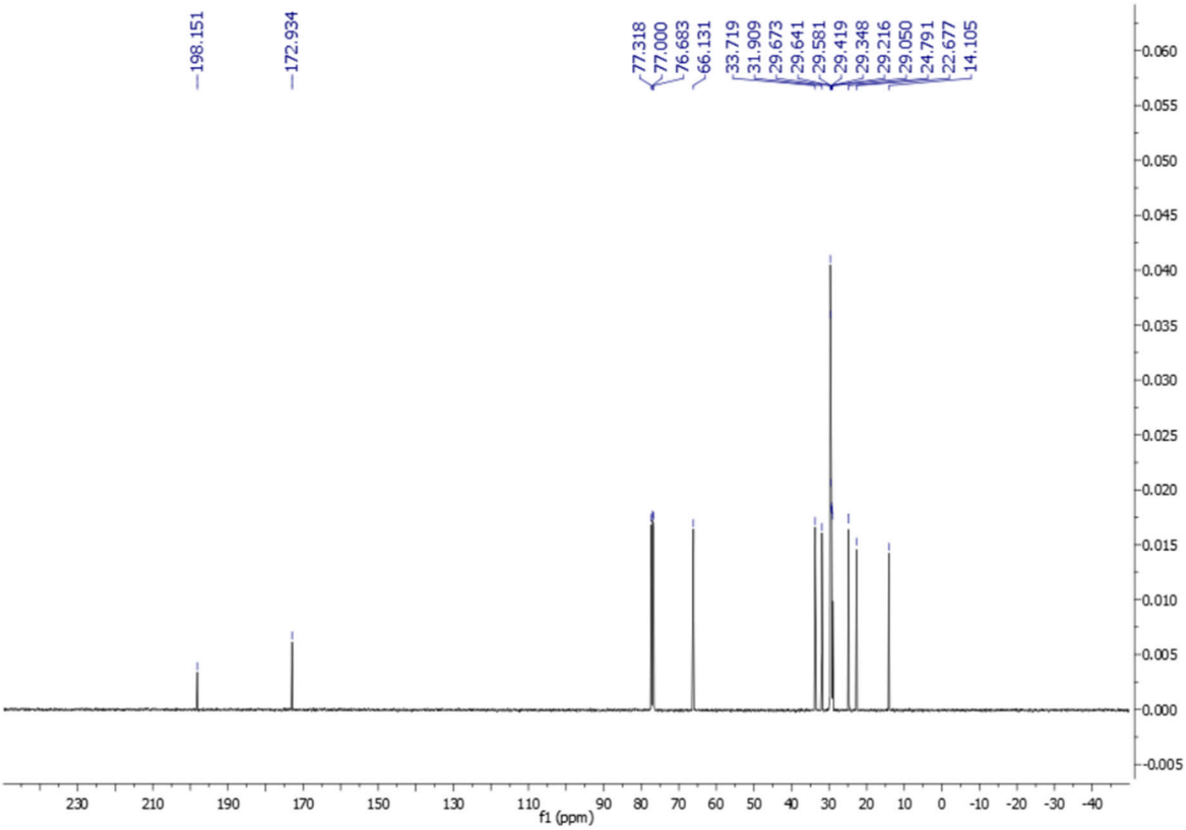

Figure S2. ^{13}C NMR spectrum of 1,3-dipalmitoyloxypropan-2-one (**2**)

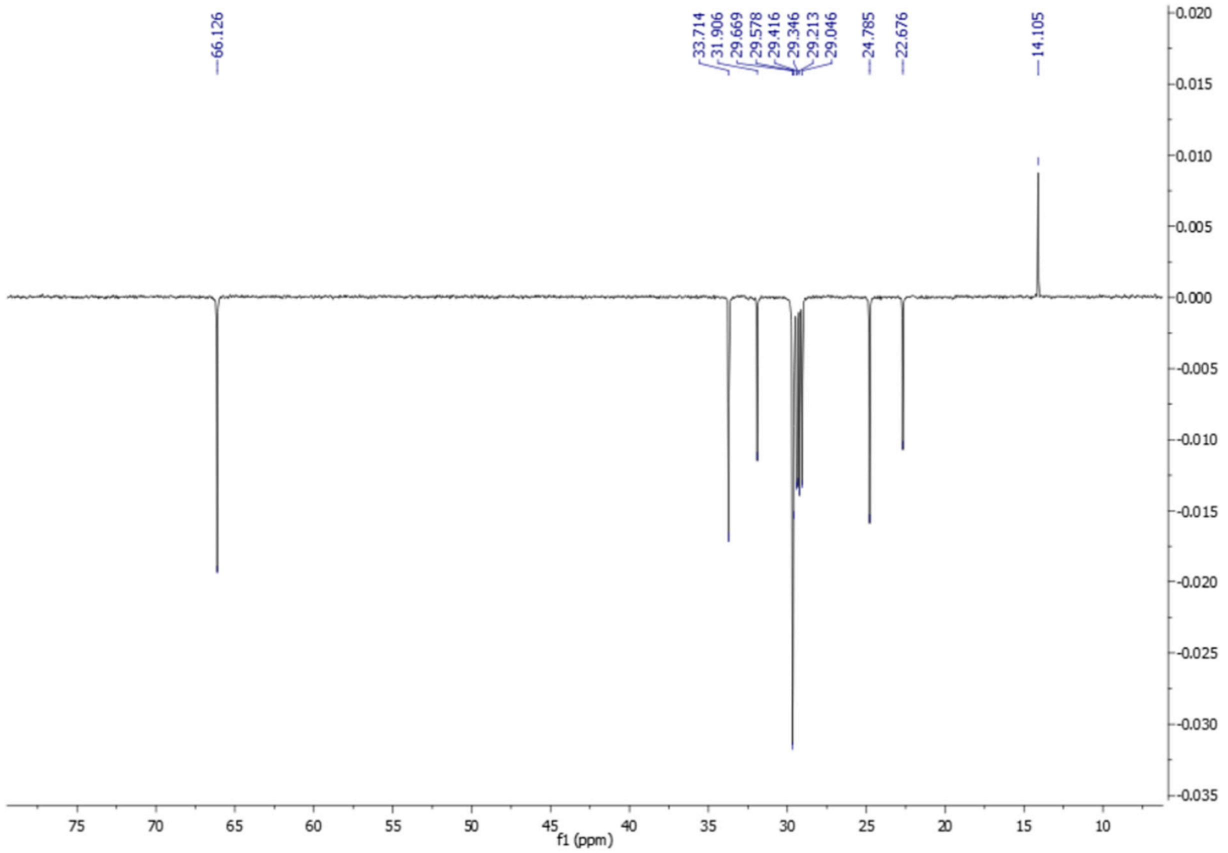

Figure S3. DEPT 135 spectrum of 1,3-dipalmitoyloxypropan-2-one (**2**)

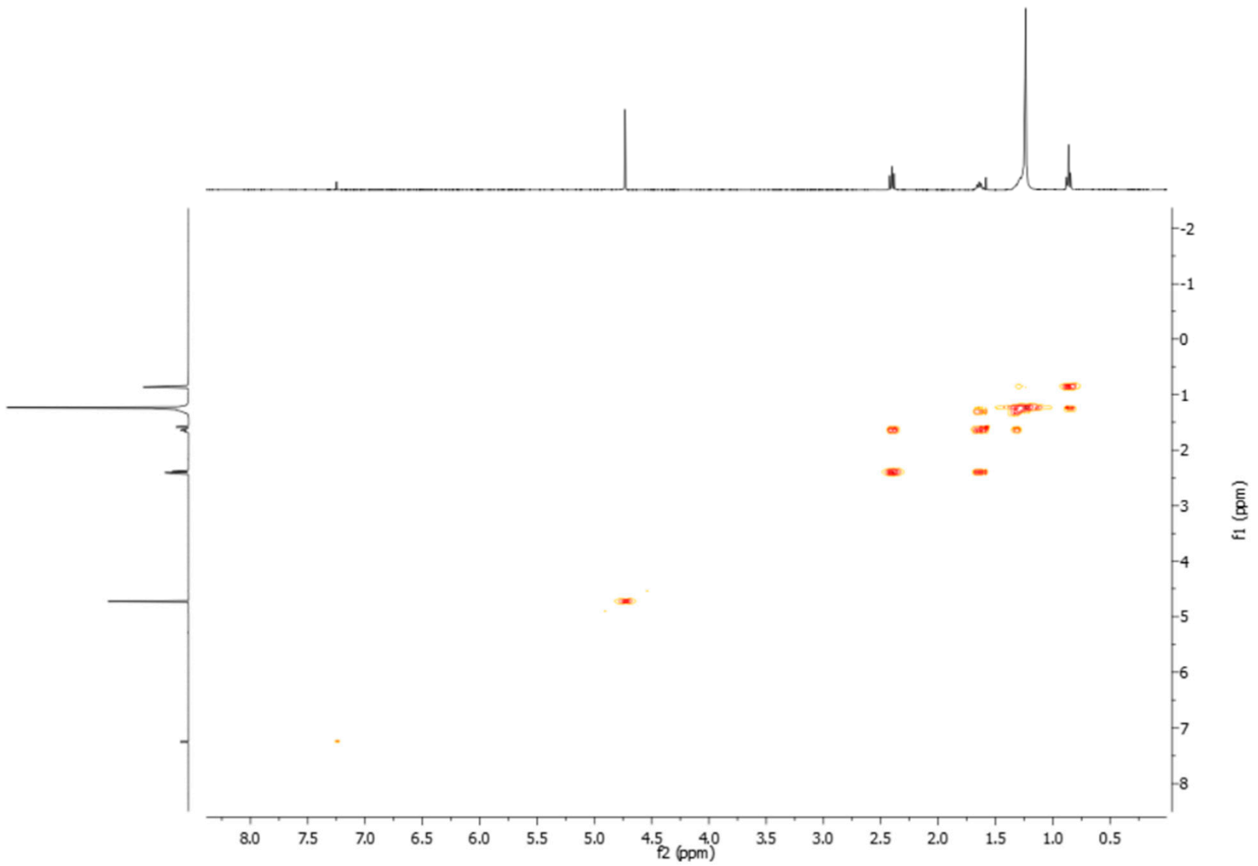

Figure S4. COSY spectrum of 1,3-dipalmitoyloxypropan-2-one (**2**)

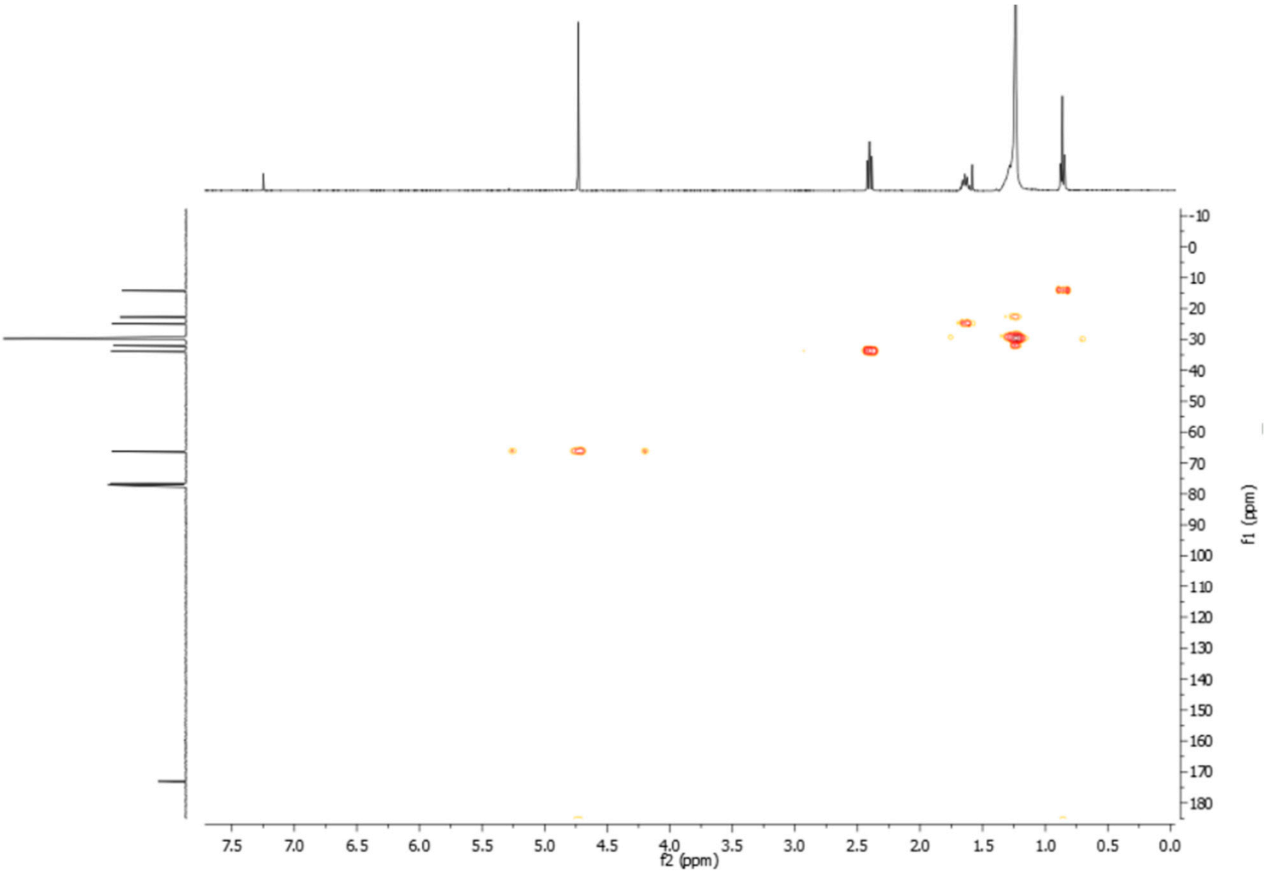

Figure S5. HMQC spectrum of 1,3-dipalmitoyloxypropan-2-one (**2**)

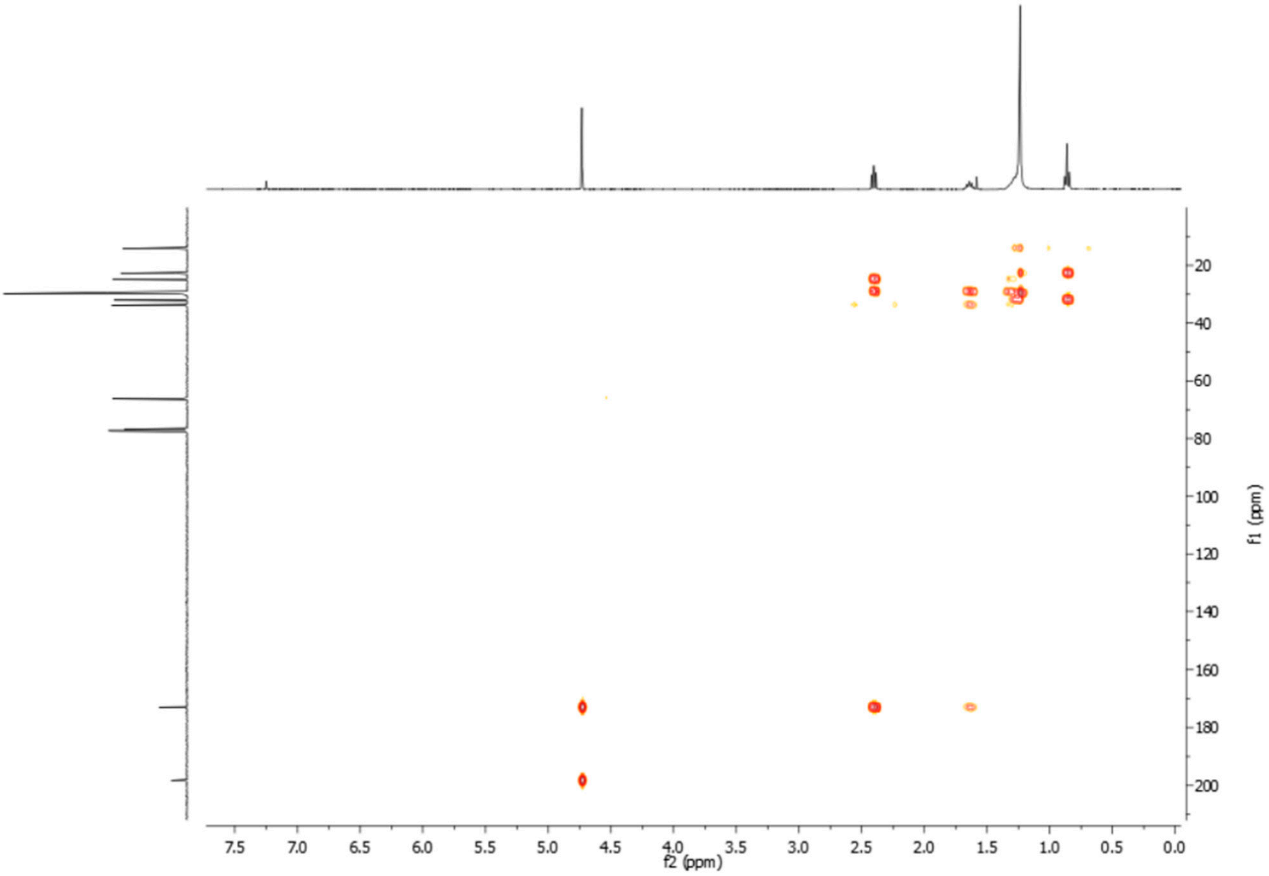

Figure S6. HMBC spectrum of 1,3-dipalmitoyloxypropan-2-one (**2**)

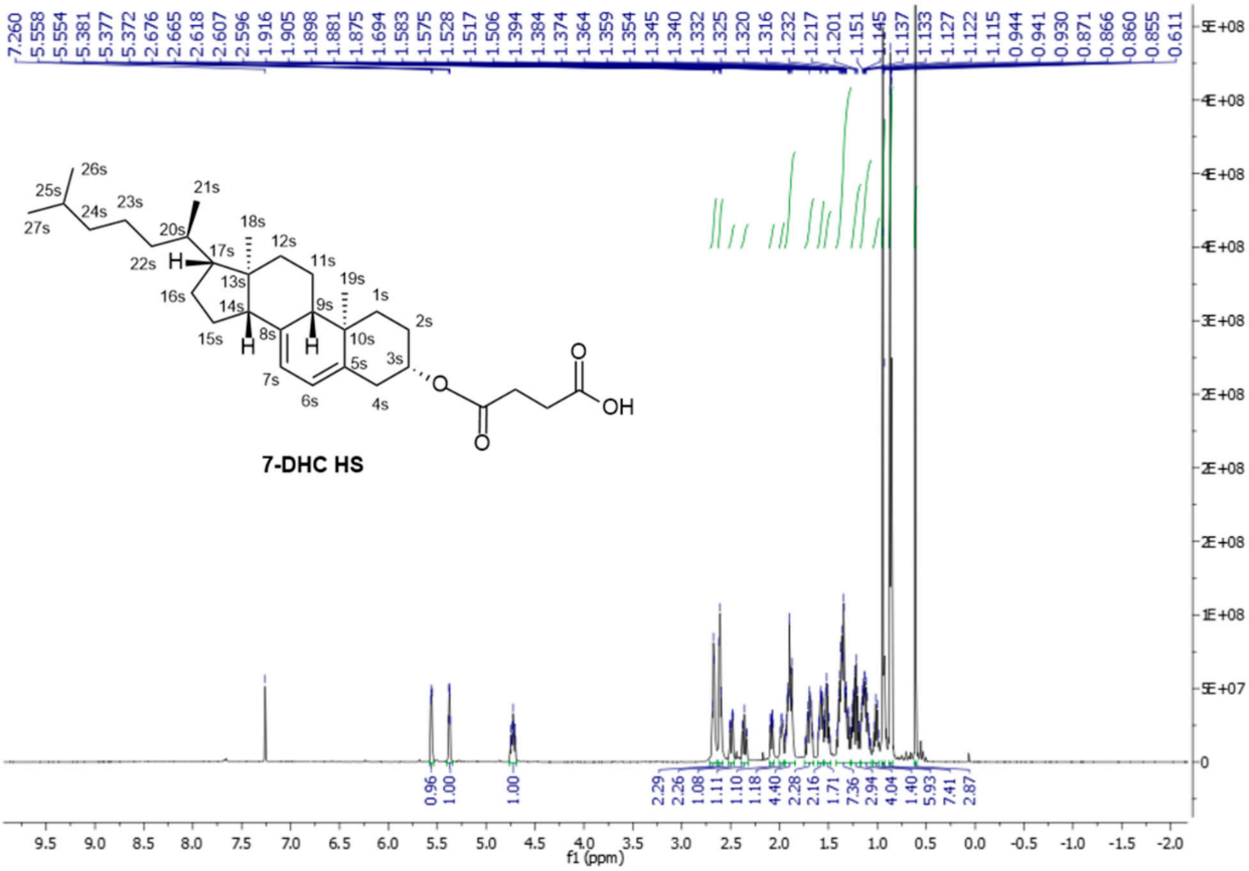

Figure S7. ^{1}H NMR spectrum of 7-dehydrocholesterol hemisuccinate (**7-DHC HS**)

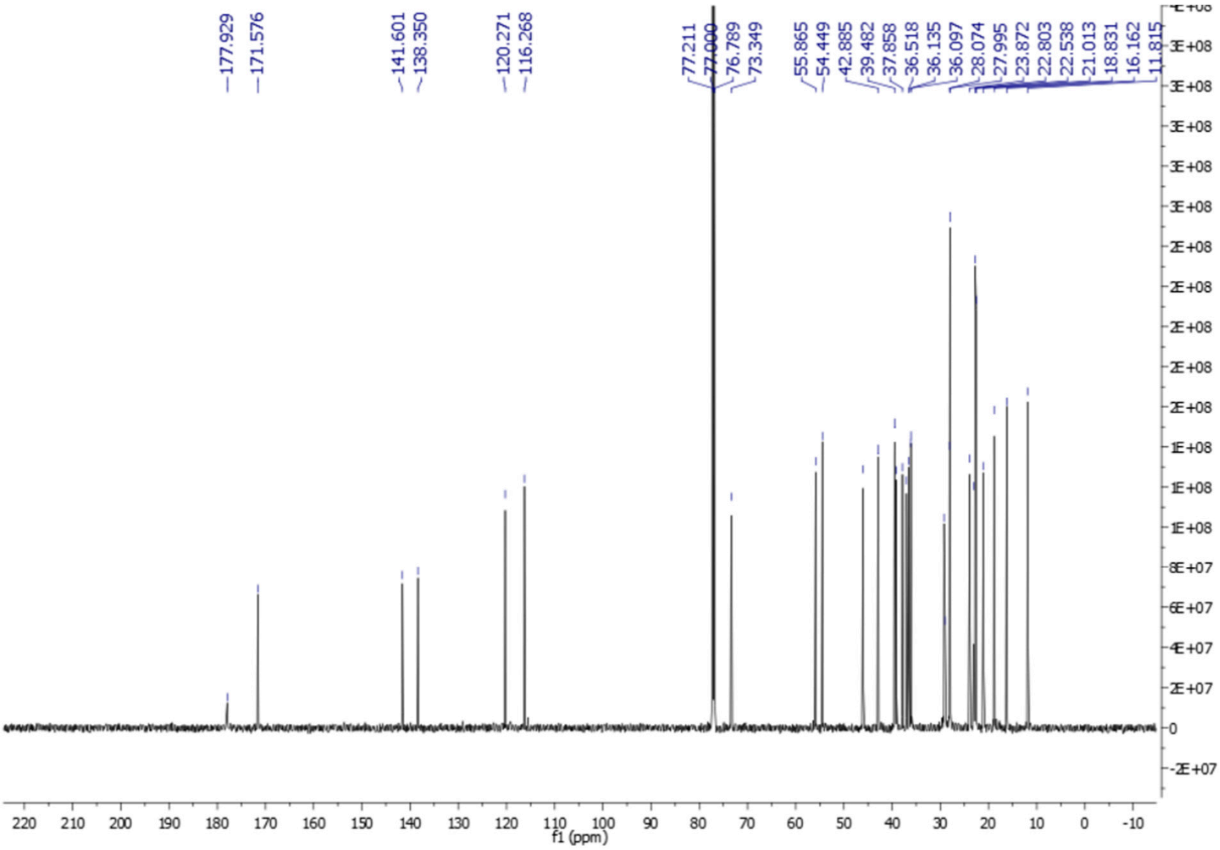

Figure S8. ^{13}C NMR spectrum of 7-dehydrocholesterol hemisuccinate (**7-DHC HS**)

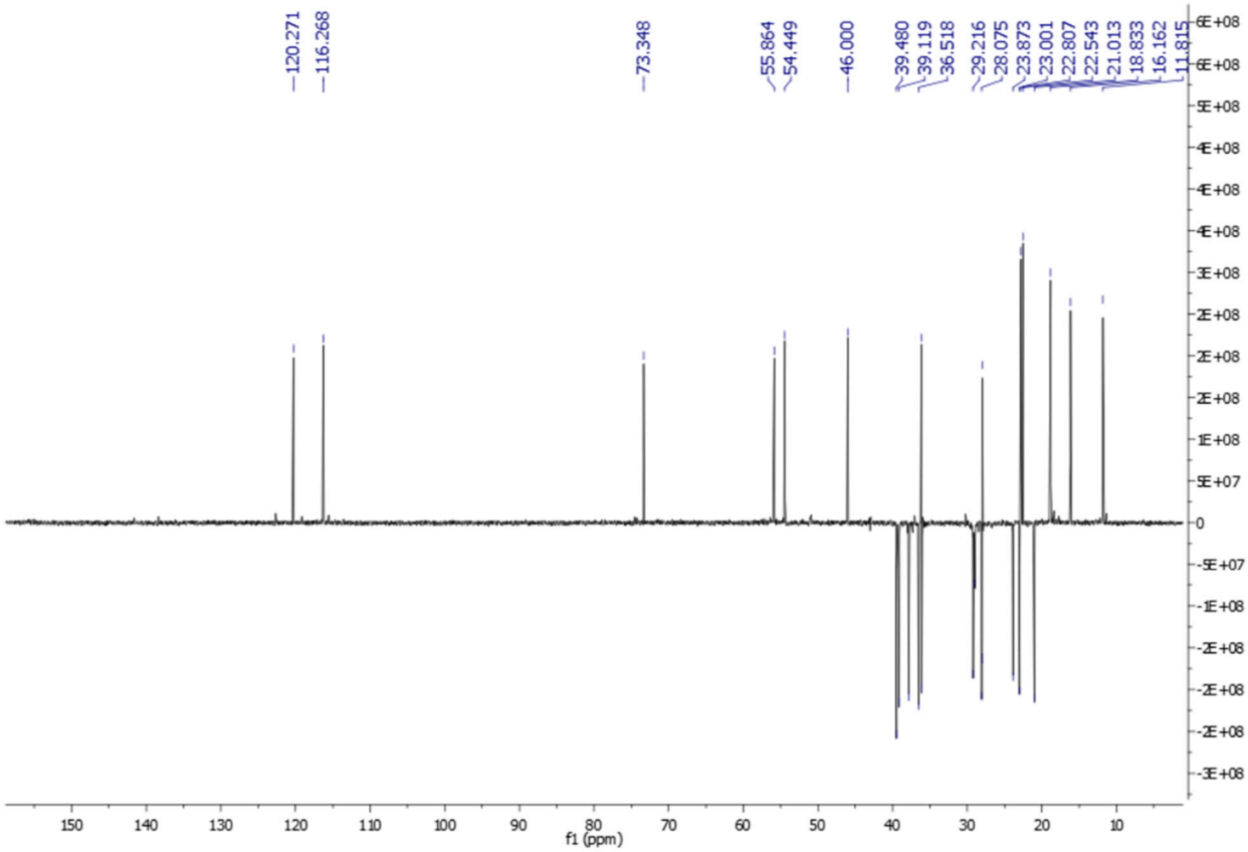

Figure S9. DEPT 135 NMR spectrum of 7-dehydrocholesterol hemisuccinate (**7-DHC HS**)

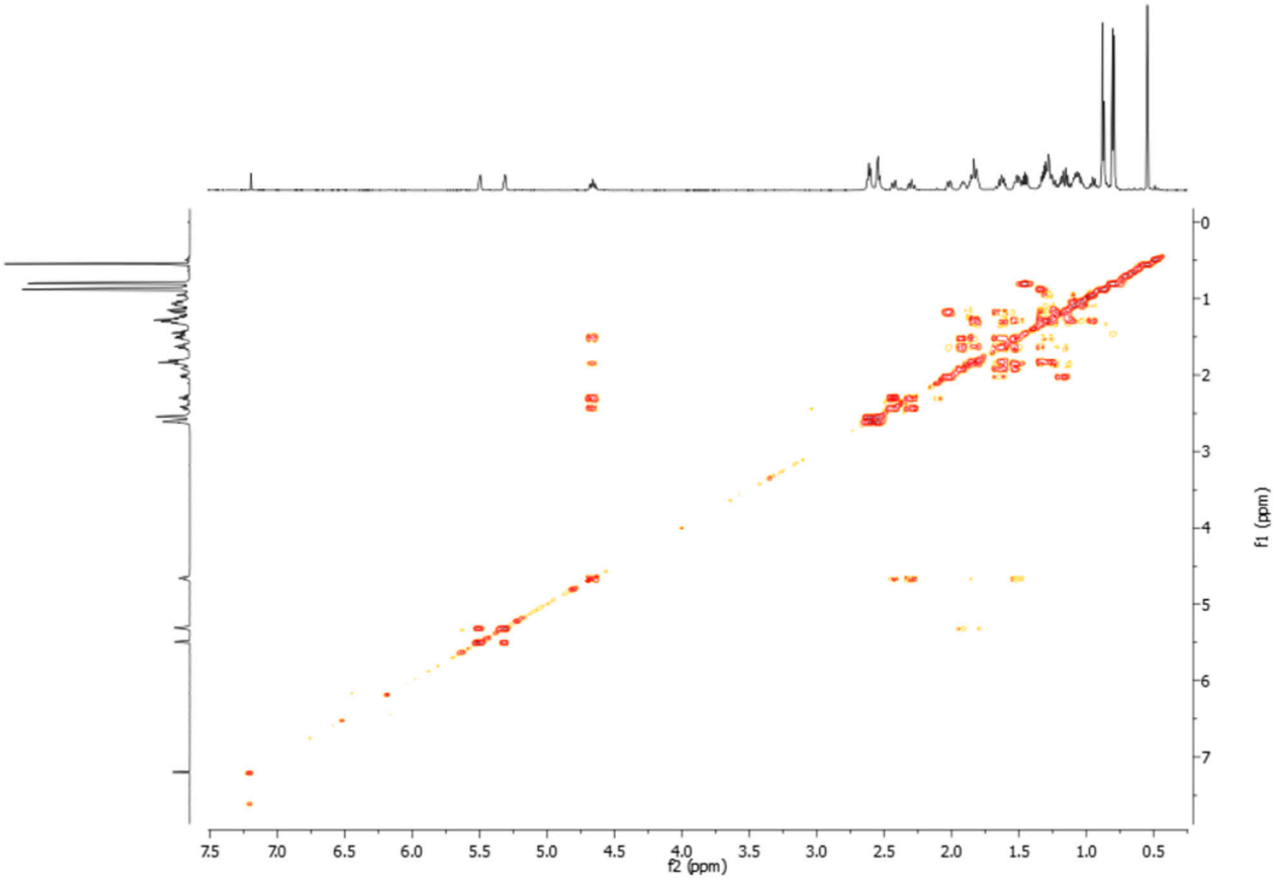

Figure S10. COSY spectrum of 7-dehydrocholesterol hemisuccinate (**7-DHC HS**)

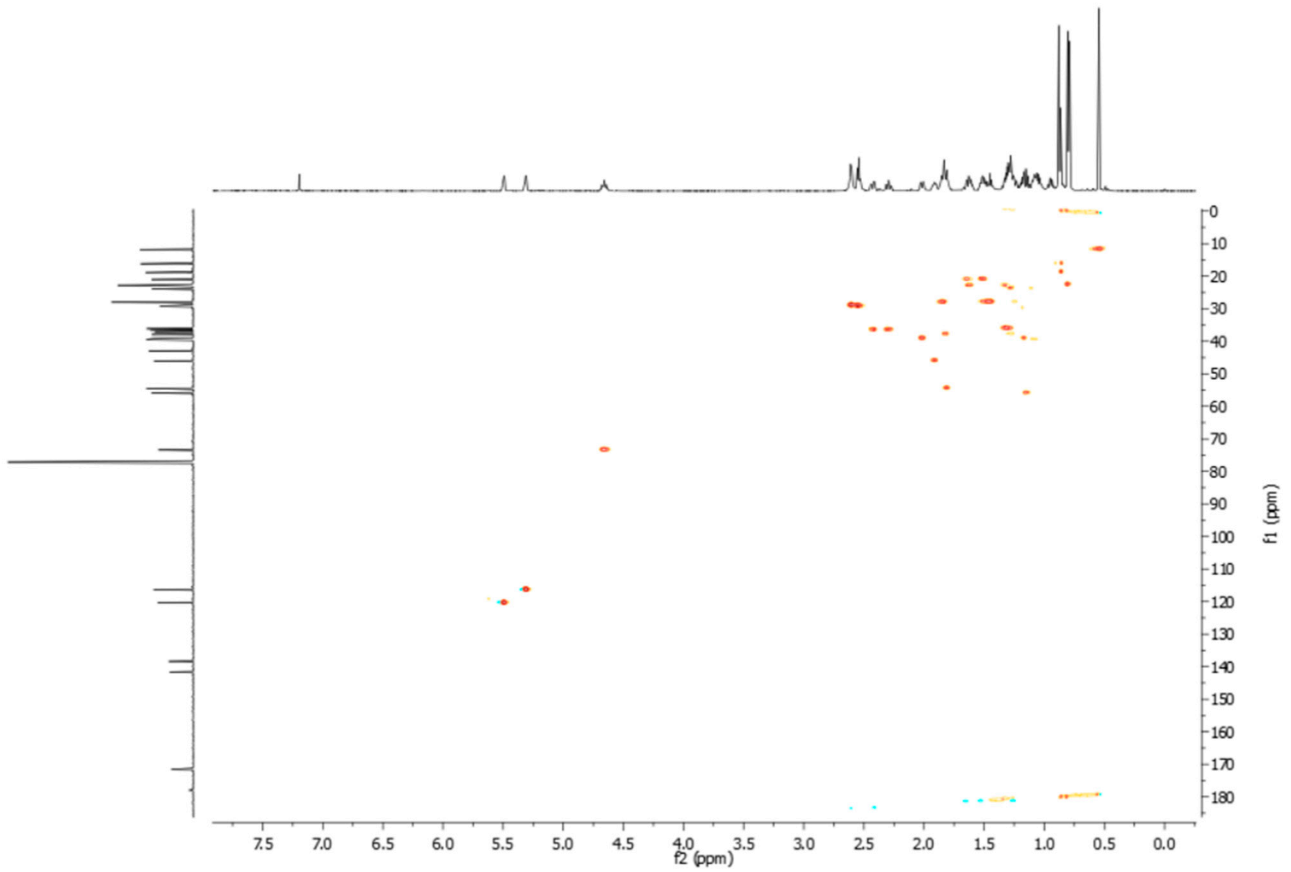

Figure S11. HMQC spectrum of 7-dehydrocholesterol hemisuccinate (**7-DHC HS**)

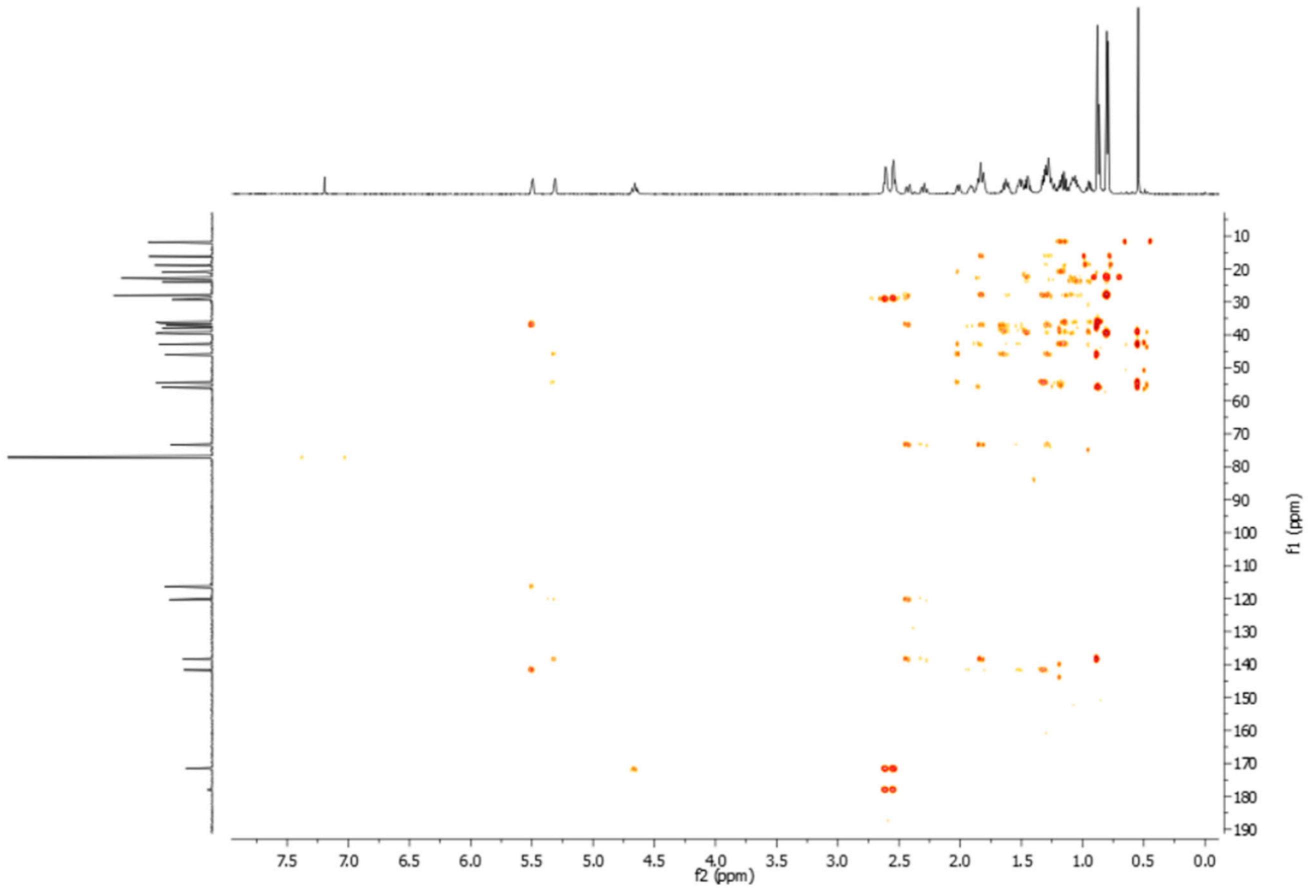

Figure S12. HMBC spectrum of 7-dehydrocholesterol hemisuccinate (**7-DHC HS**)

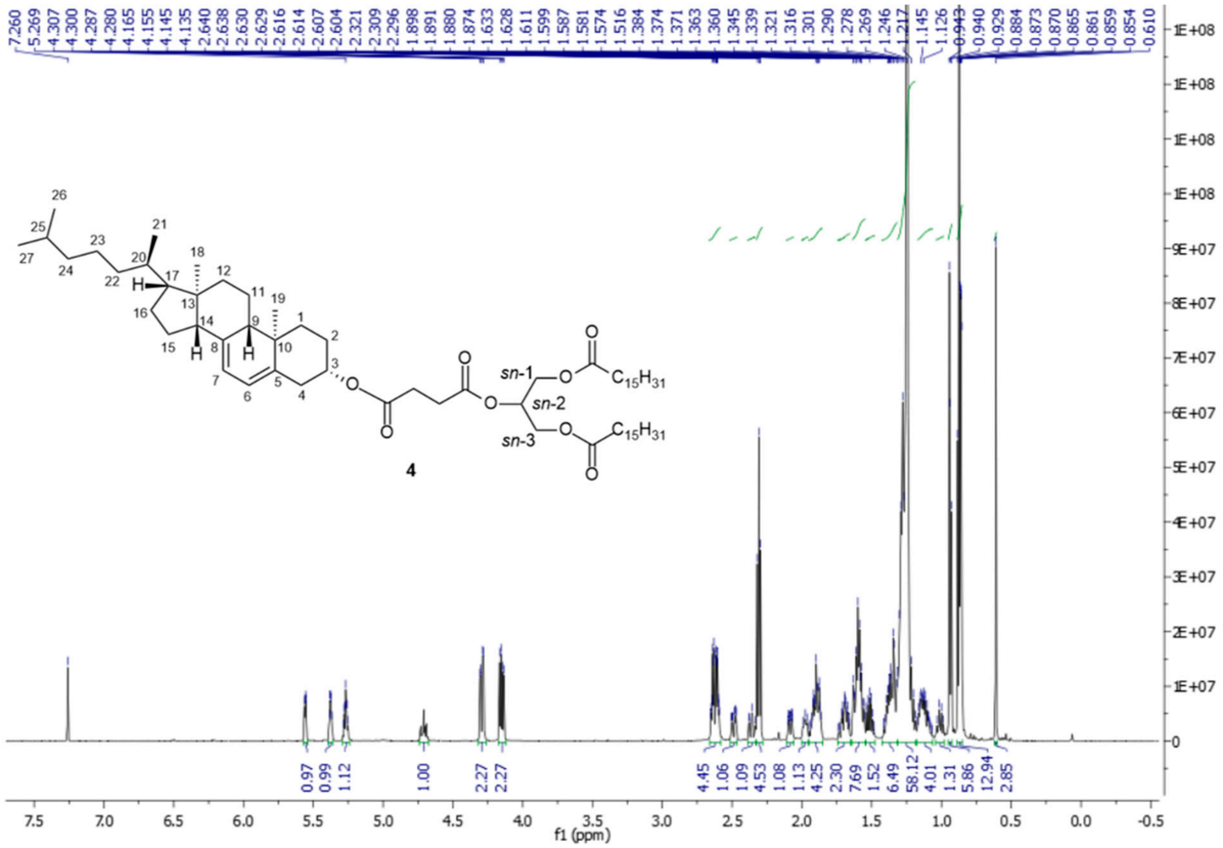

Figure S13. ^{1}H NMR spectrum of 1,3-dipalmitoyl-2-(7-dehydrocholestyrylsuccinoyl)glycerol (**4**)

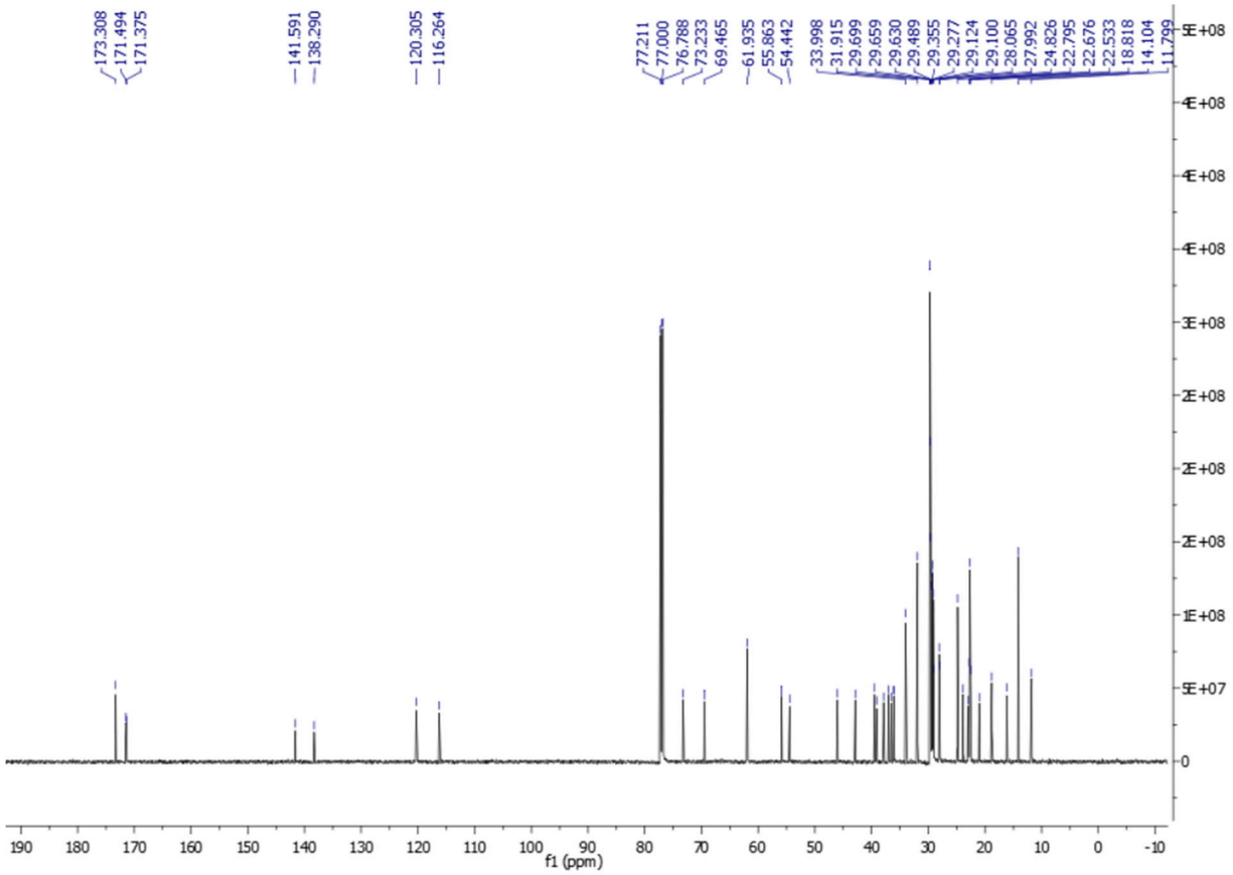

Figure S14. ^{13}C NMR spectrum of 1,3-dipalmitoyl-2-(7-dehydrocholestyrylsuccinoyl)glycerol (**4**)

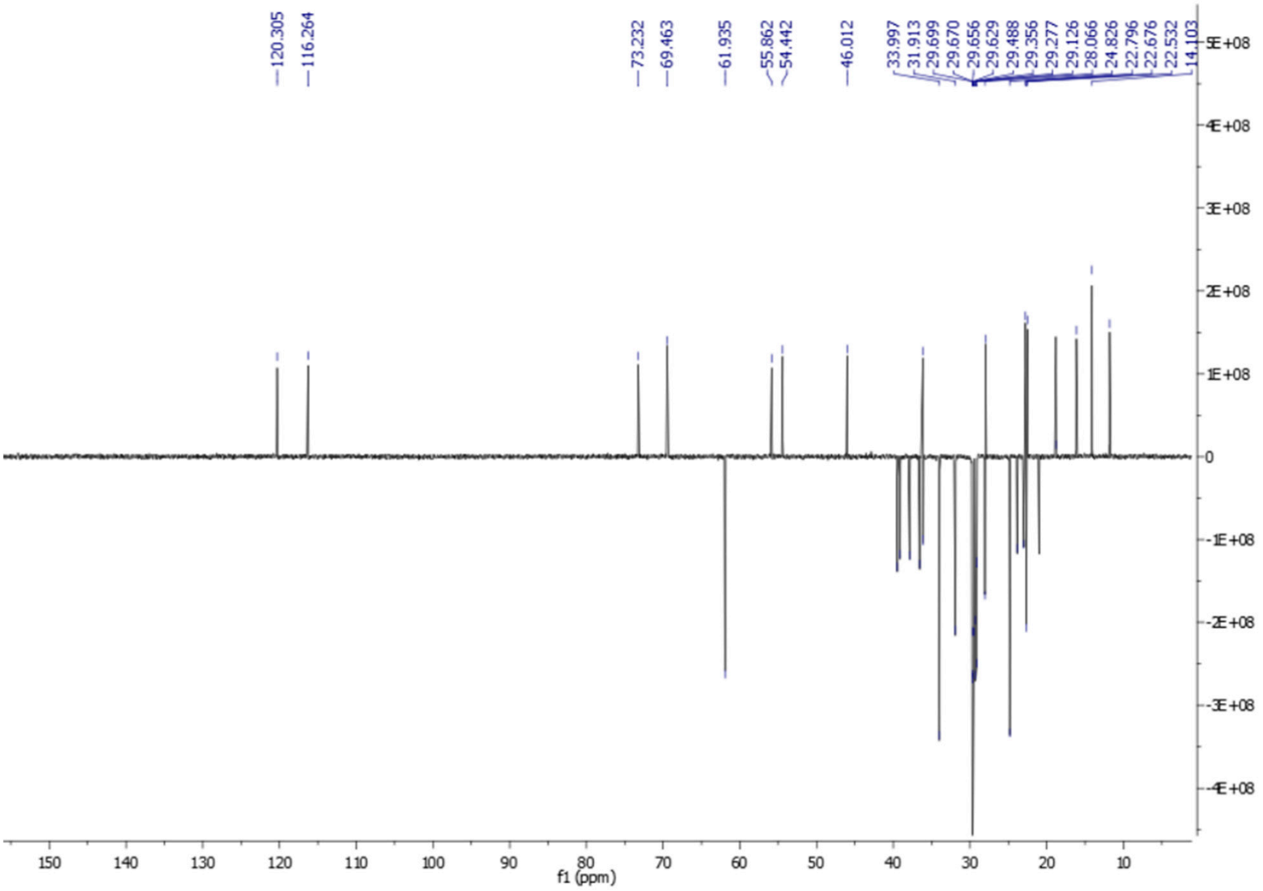

Figure S15. DEPT 135 spectrum of 1,3-dipalmitoyl-2-(7-dehydrocholestyrylsuccinoyl)glycerol (**4**)

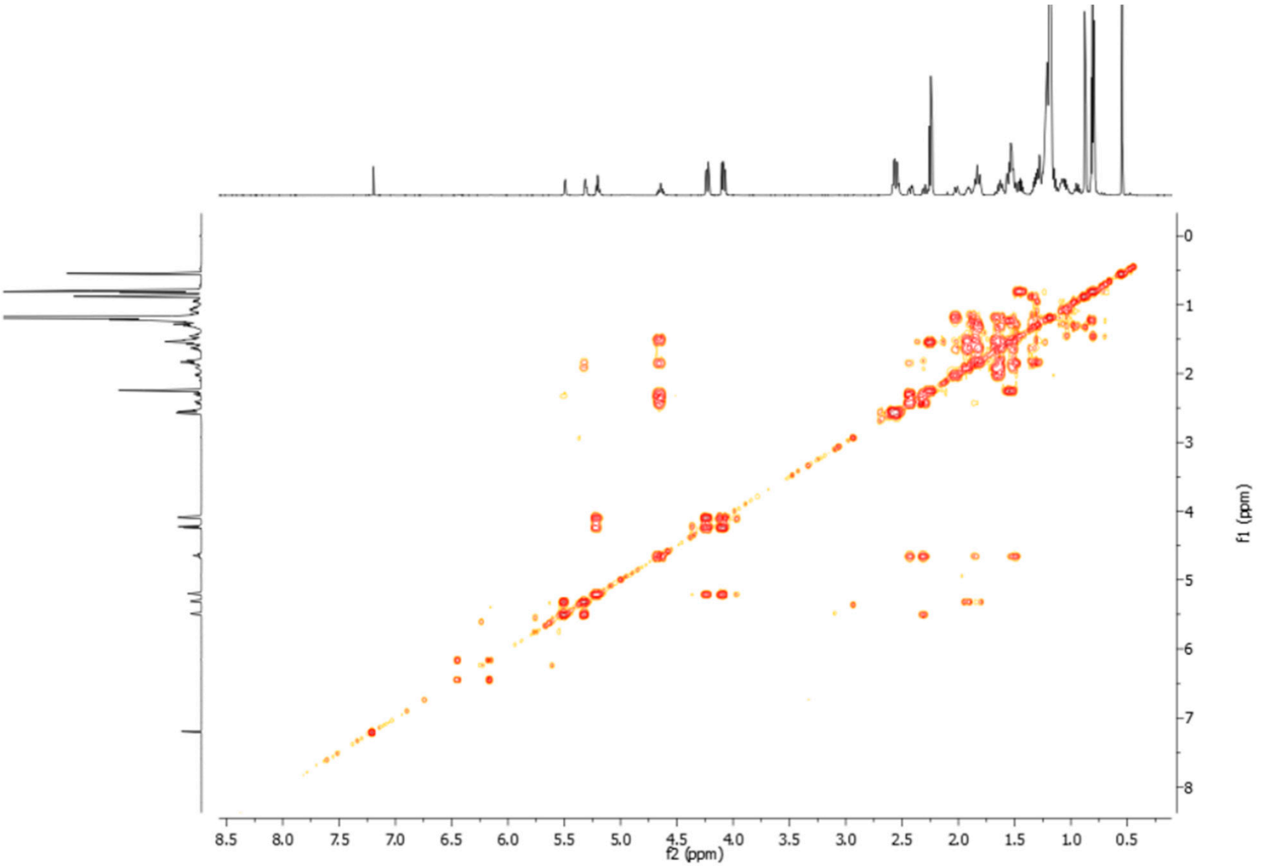

Figure S16. COSY spectrum of 1,3-dipalmitoyl-2-(7-dehydrocholestyrylsuccinoyl)glycerol (**4**)

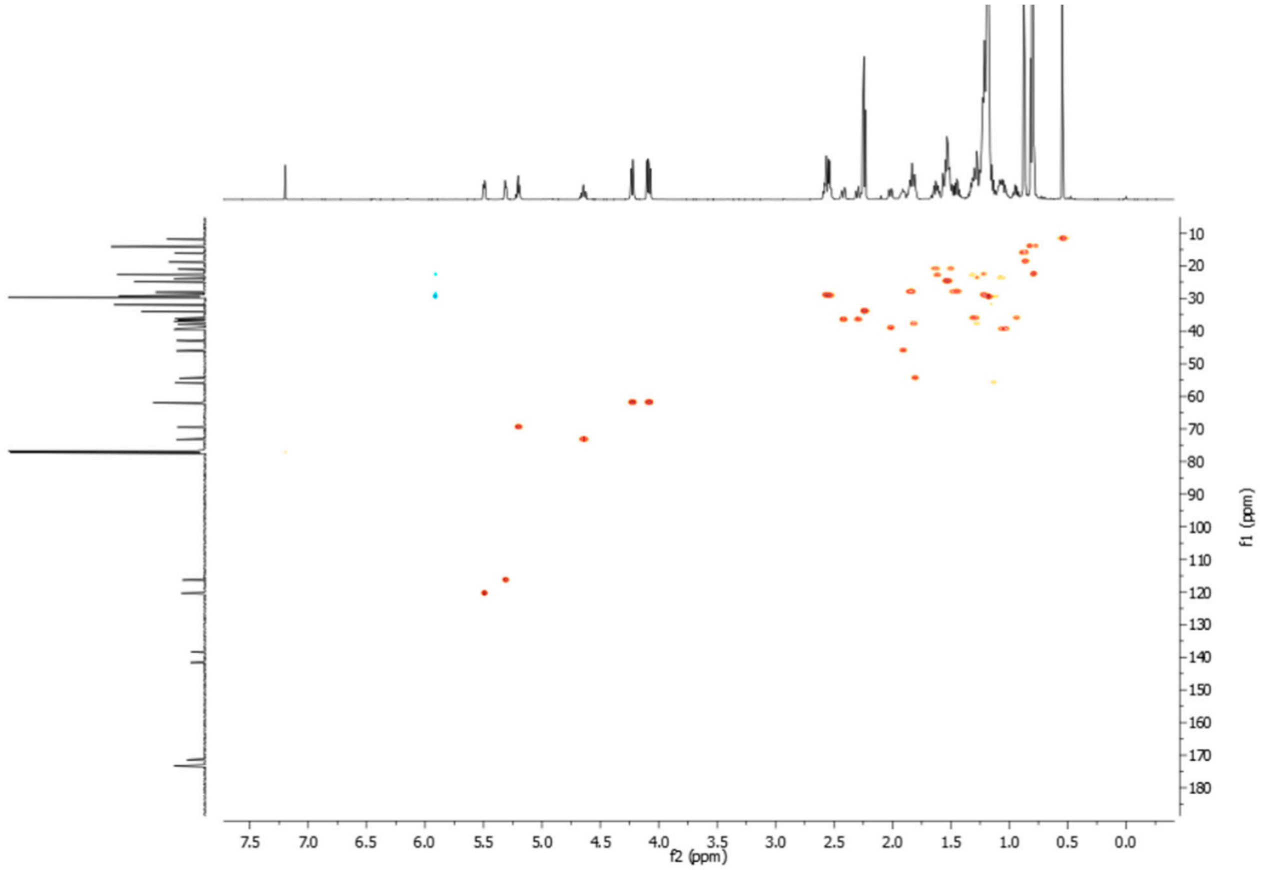

Figure S17. HMQC spectrum of 1,3-dipalmitoyl-2-(7-dehydrocholestyrylsuccinoyl)glycerol (**4**)

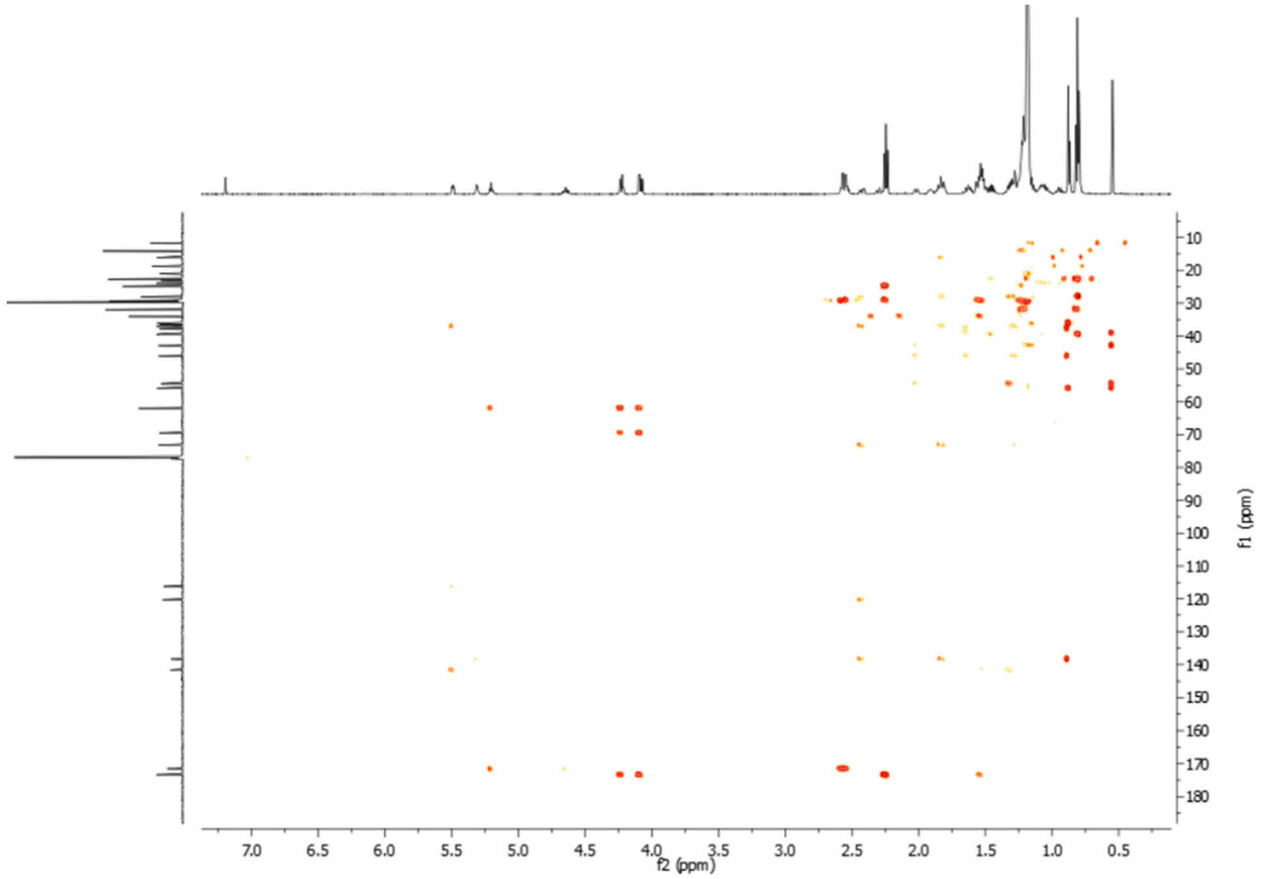

Figure S18. HMBC spectrum of 1,3-dipalmitoyl-2-(7-dehydrocholestyrylsuccinoyl)glycerol (**4**)

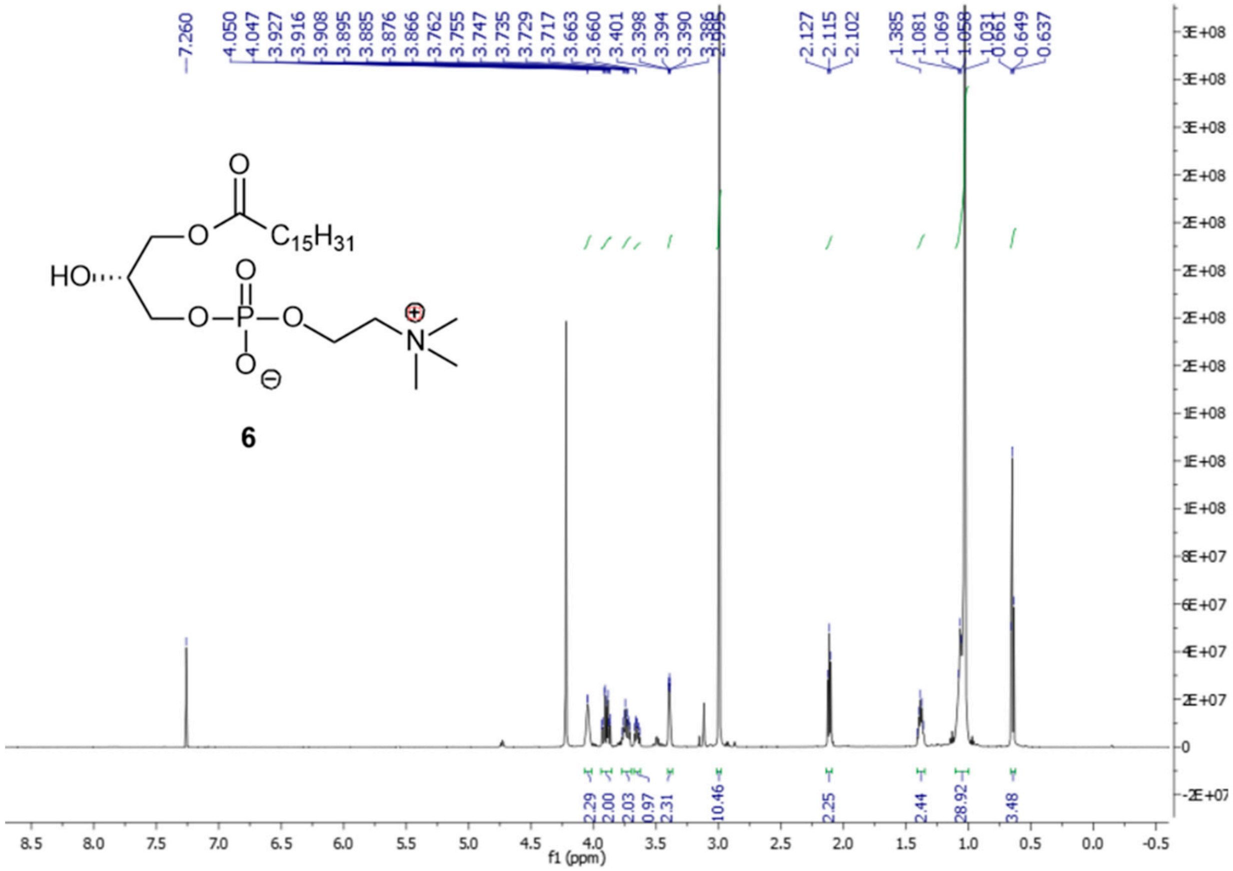

Figure S19. ^{1}H NMR spectrum of 1-palmitoyl-*sn*-glycero-3-phosphocholine (**6**)

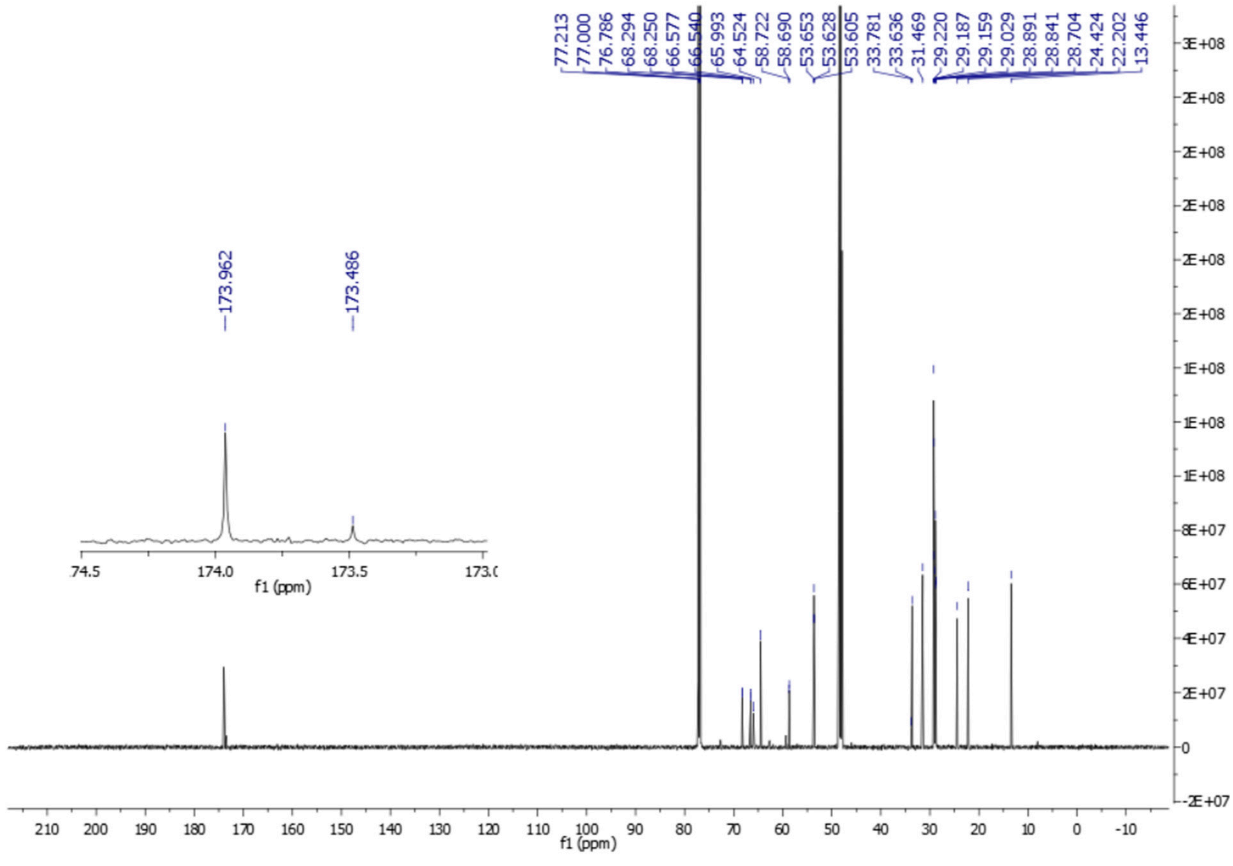

Figure S20. ^{13}C NMR spectrum of 1-palmitoyl-*sn*-glycero-3-phosphocholine (**6**)

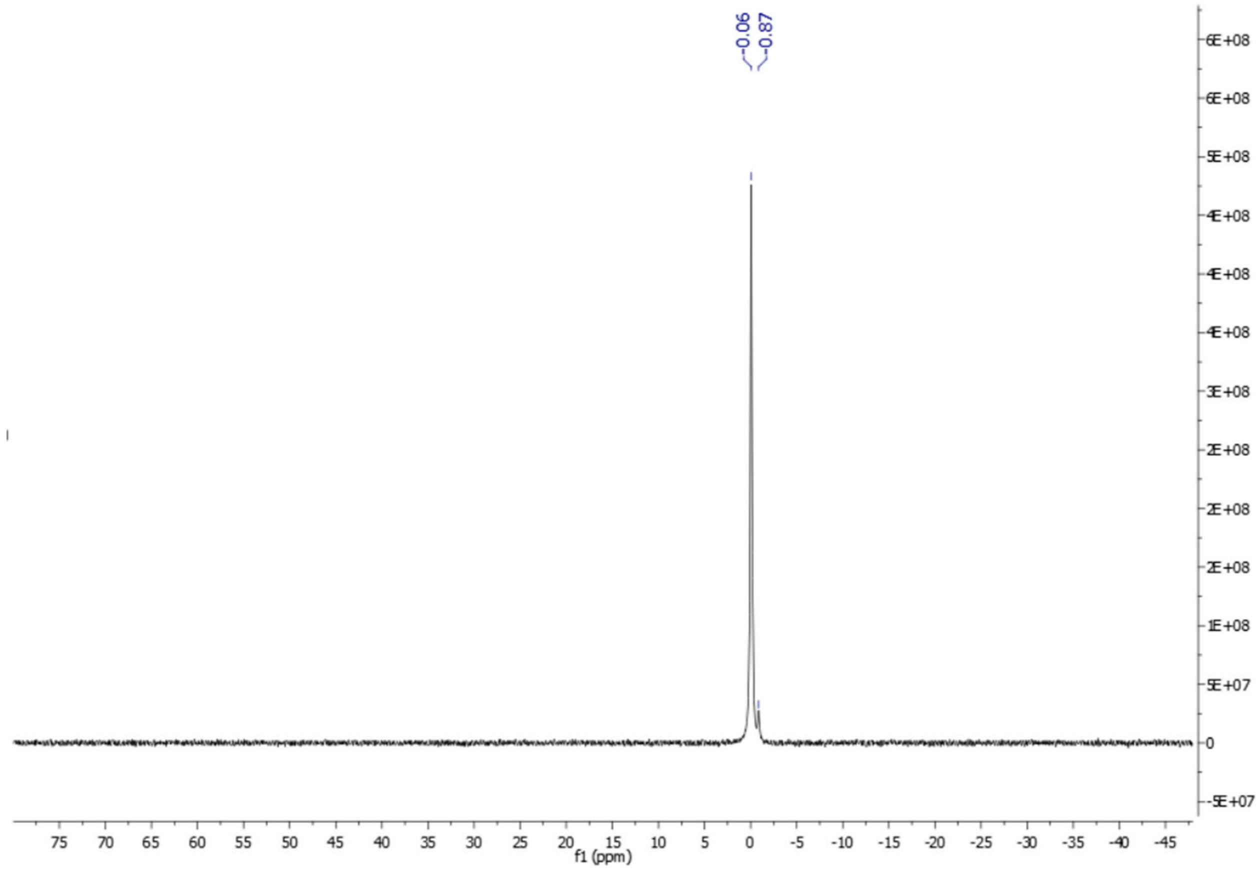

Figure S21. ^{31}P NMR spectrum of 1-palmitoyl-*sn*-glycero-3-phosphocholine (**6**)

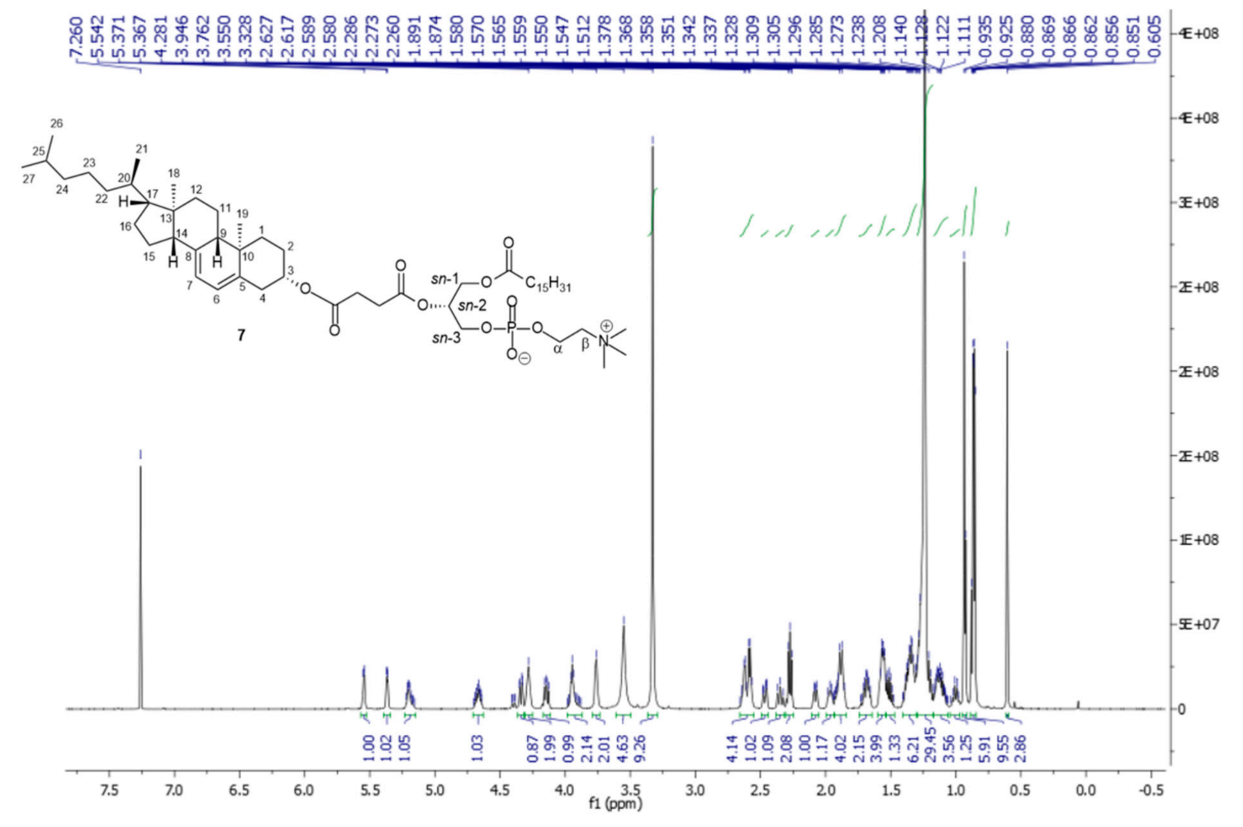

Figure S22. ^{1}H NMR spectrum of 1-palmitoyl-2-(7-dehydrocholesterylsuccinoyl)-*sn*-glycero-3-phosphocholine (**7**)

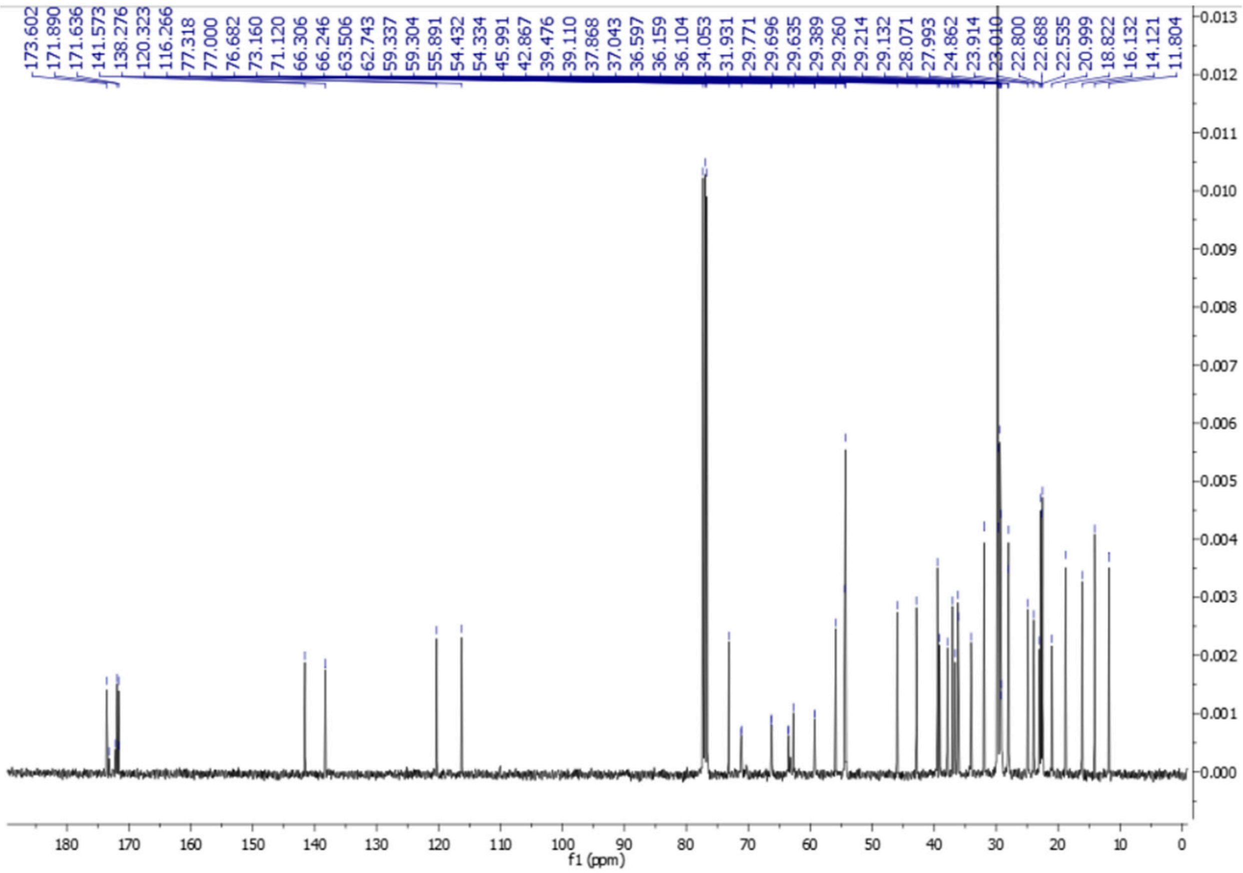

Figure S23. ^{13}C NMR spectrum of 1-palmitoyl-2-(7-dehydrocholesterylsuccinoyl)-*sn*-glycero-3-phosphocholine (**7**)

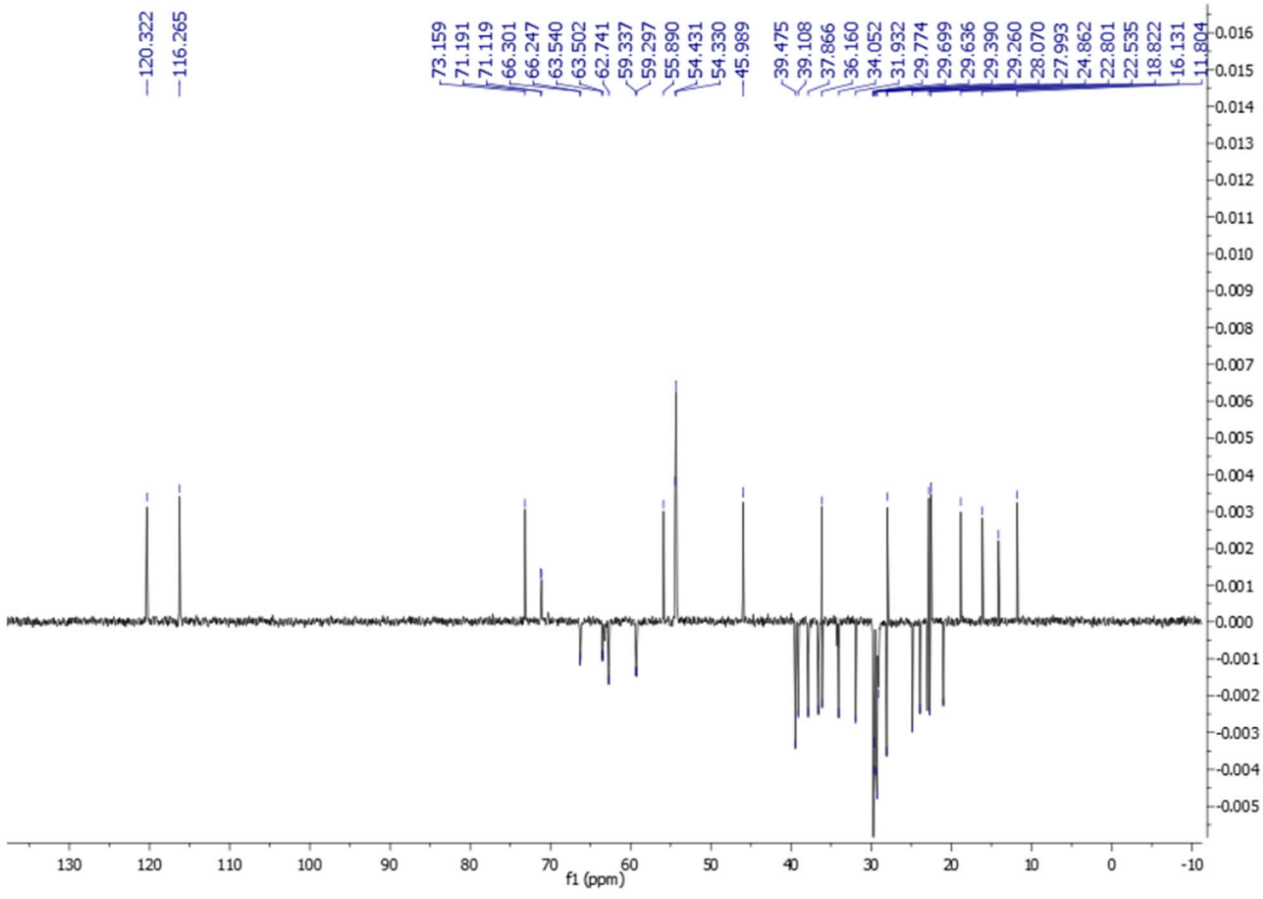

Figure S24. DEPT 135 spectrum of 1-palmitoyl-2-(7-dehydrocholesterylsuccinoyl)-*sn*-glycero-3-phosphocholine (**7**)

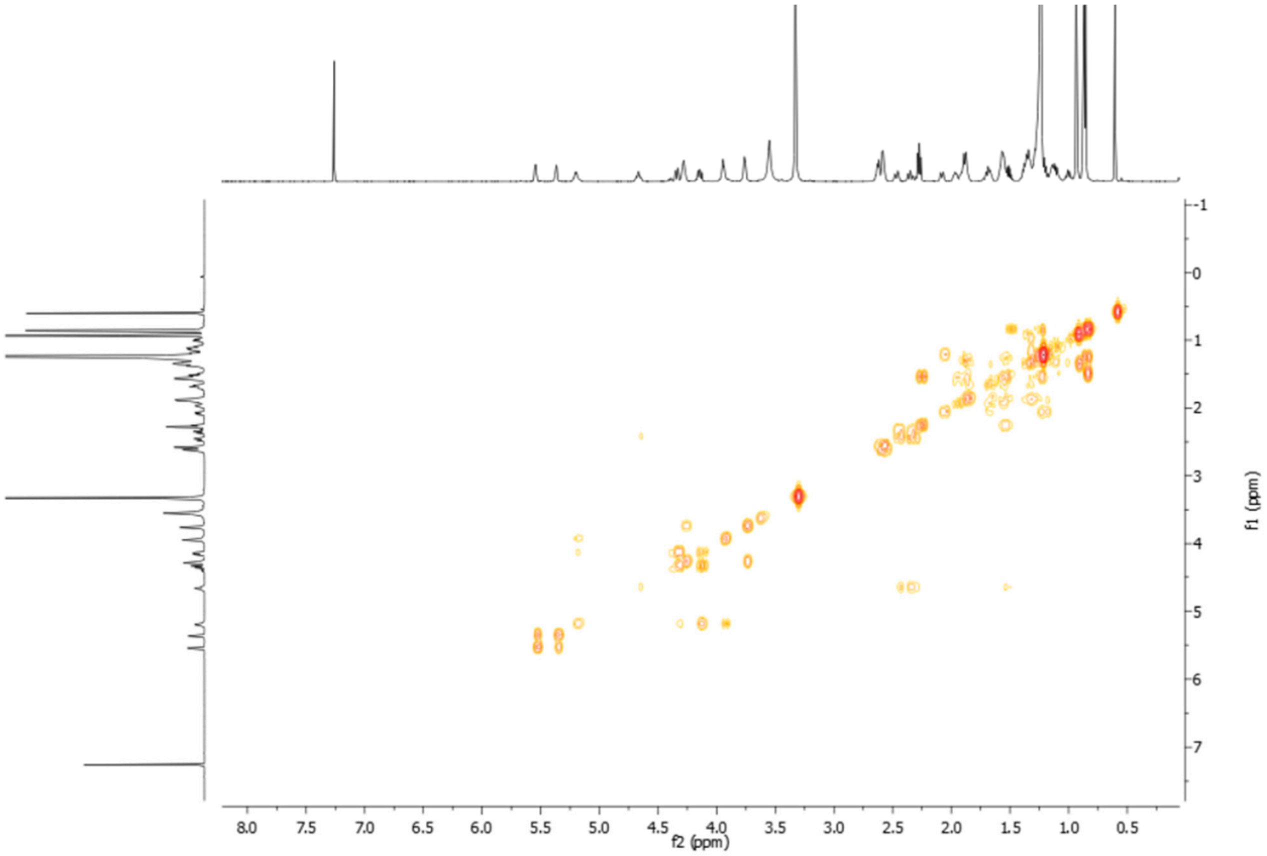

Figure S25. COSY spectrum of 1-palmitoyl-2-(7-dehydrocholesterylsuccinoyl)-*sn*-glycero-3-phosphocholine (**7**)

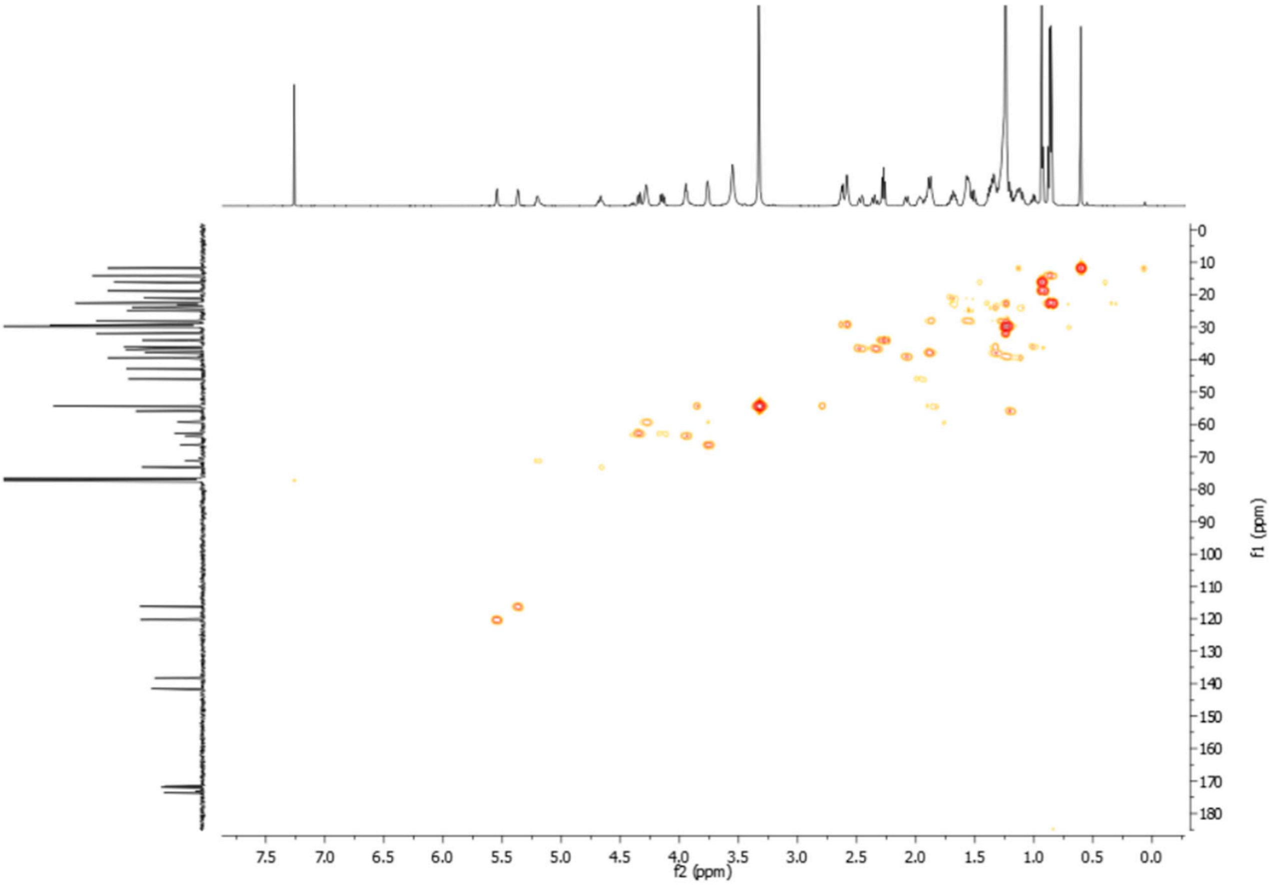

Figure S26. HMQC spectrum of 1-palmitoyl-2-(7-dehydrocholesterylsuccinoyl)-*sn*-glycero-3-phosphocholine (**7**)

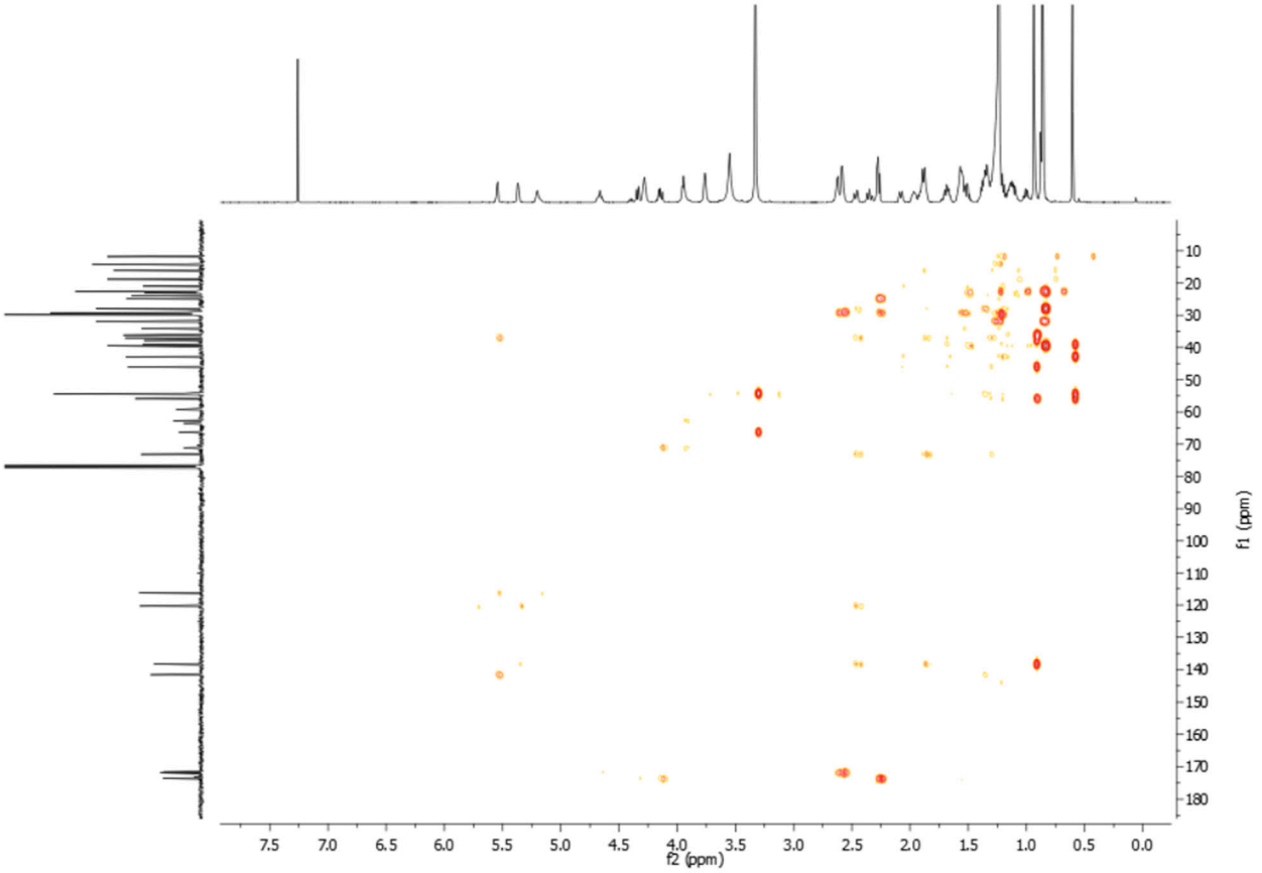

Figure S27. HMBC spectrum of 1-palmitoyl-2-(7-dehydrocholesterylsuccinoyl)-*sn*-glycero-3-phosphocholine (**7**)

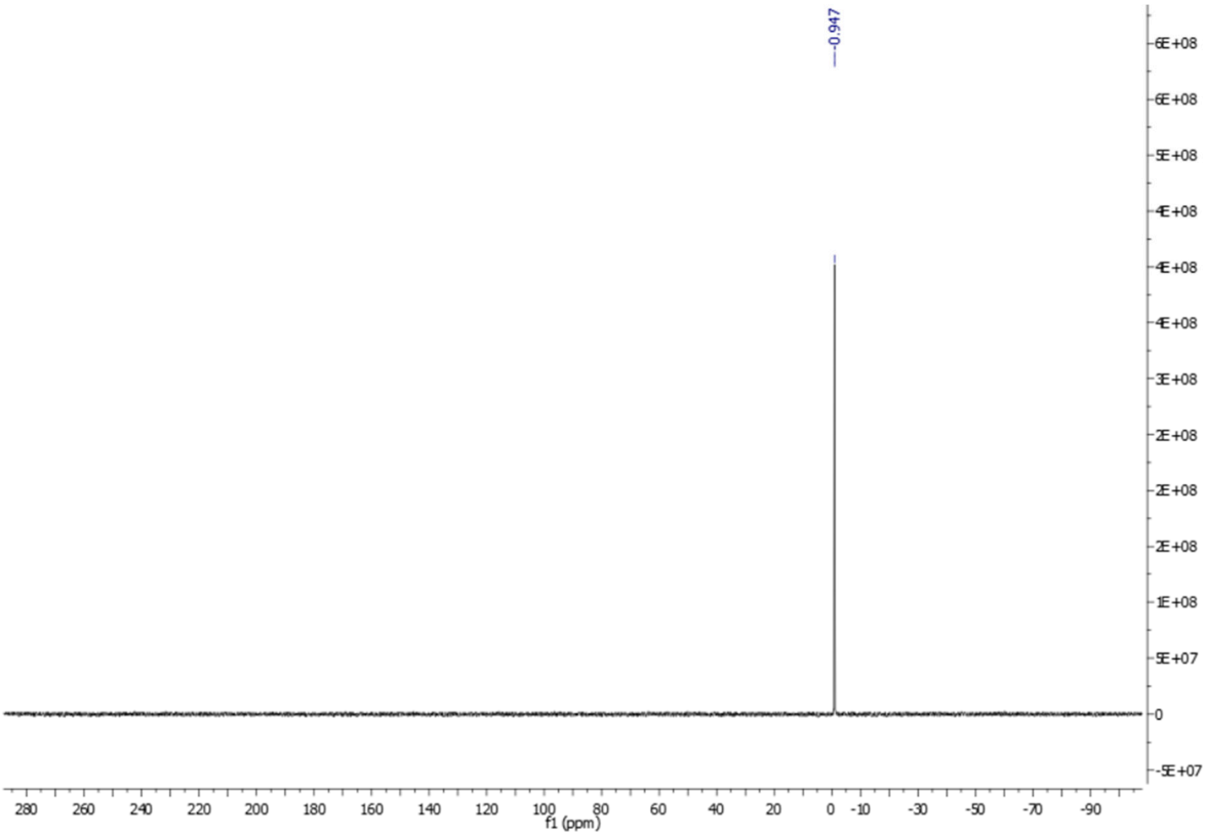

Figure S28. ^{31}P NMR spectrum of 1-palmitoyl-2-(7-dehydrocholesterylsuccinoyl)-*sn*-glycero-3-phosphocholine (**7**)

IR SPECTRA:

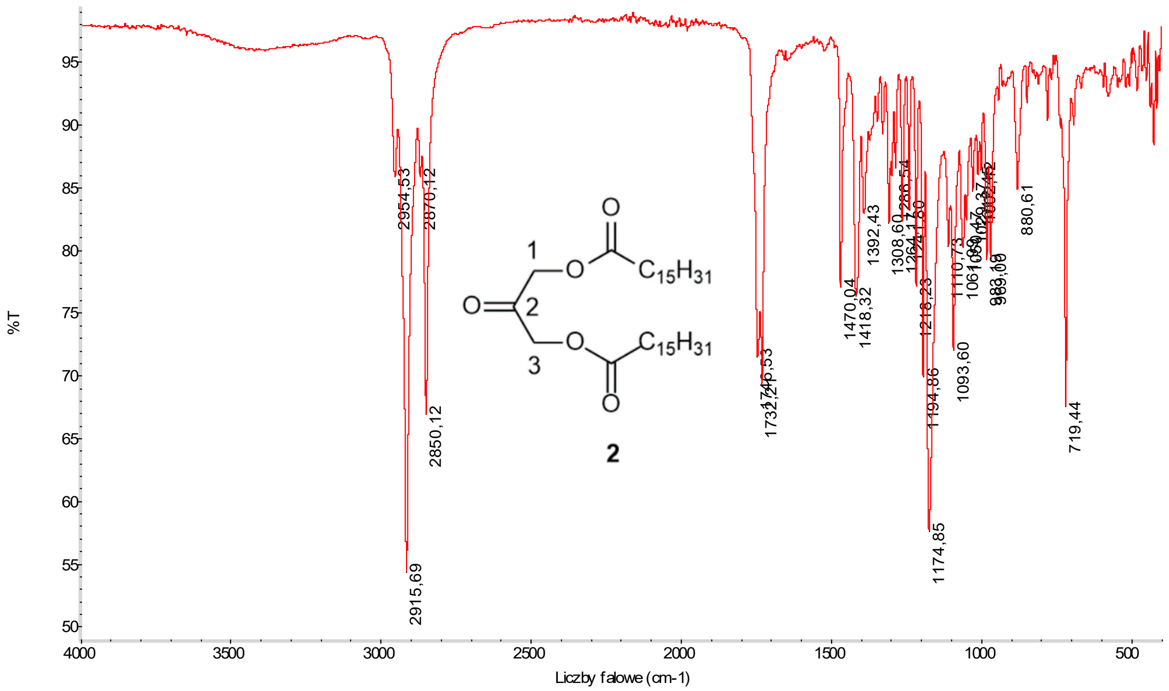

Figure S29. IR spectrum of 1,3-dipalmitoyloxypropan-2-one (**2**)

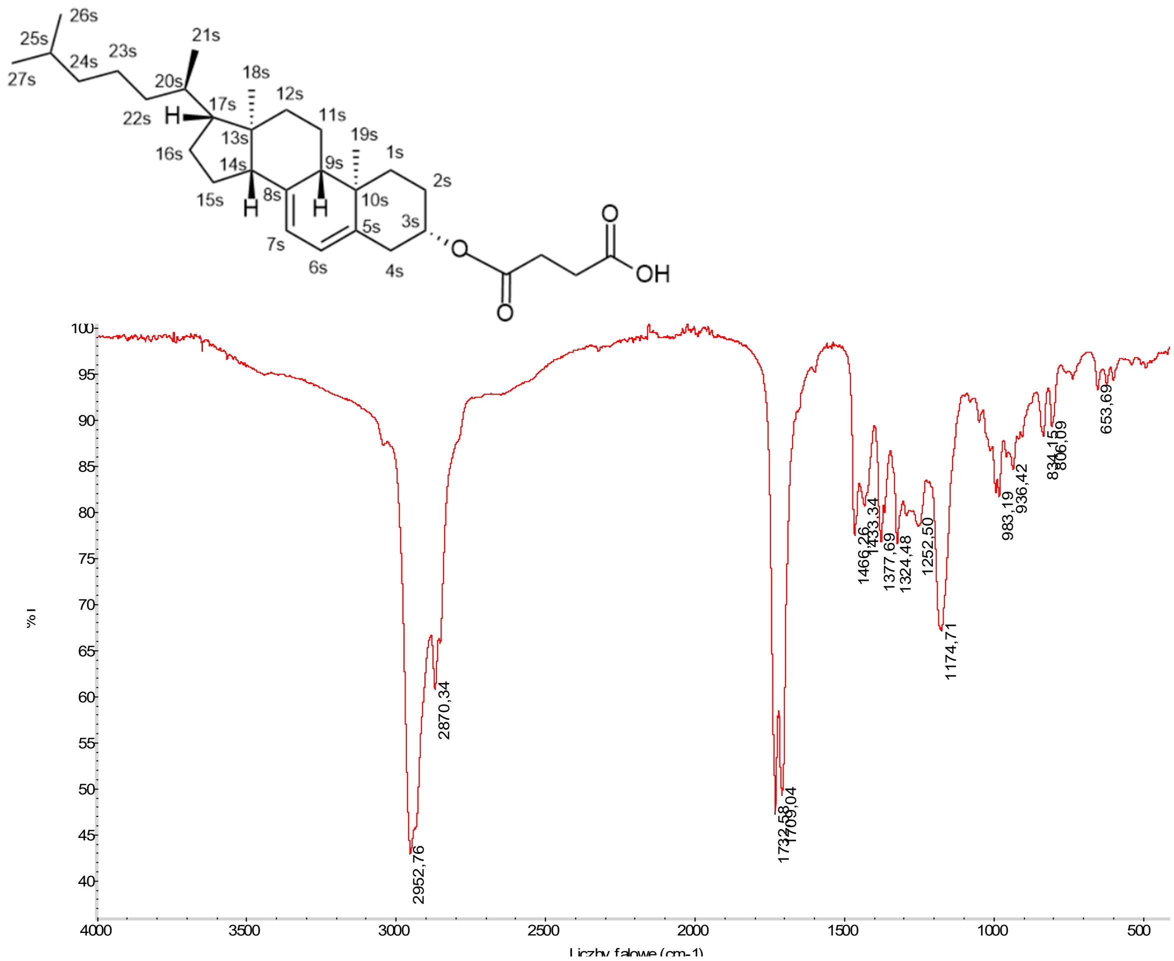

Figure S30. IR spectrum of 7-dehydrocholesterol hemisuccinate (**7-DHC HS**)

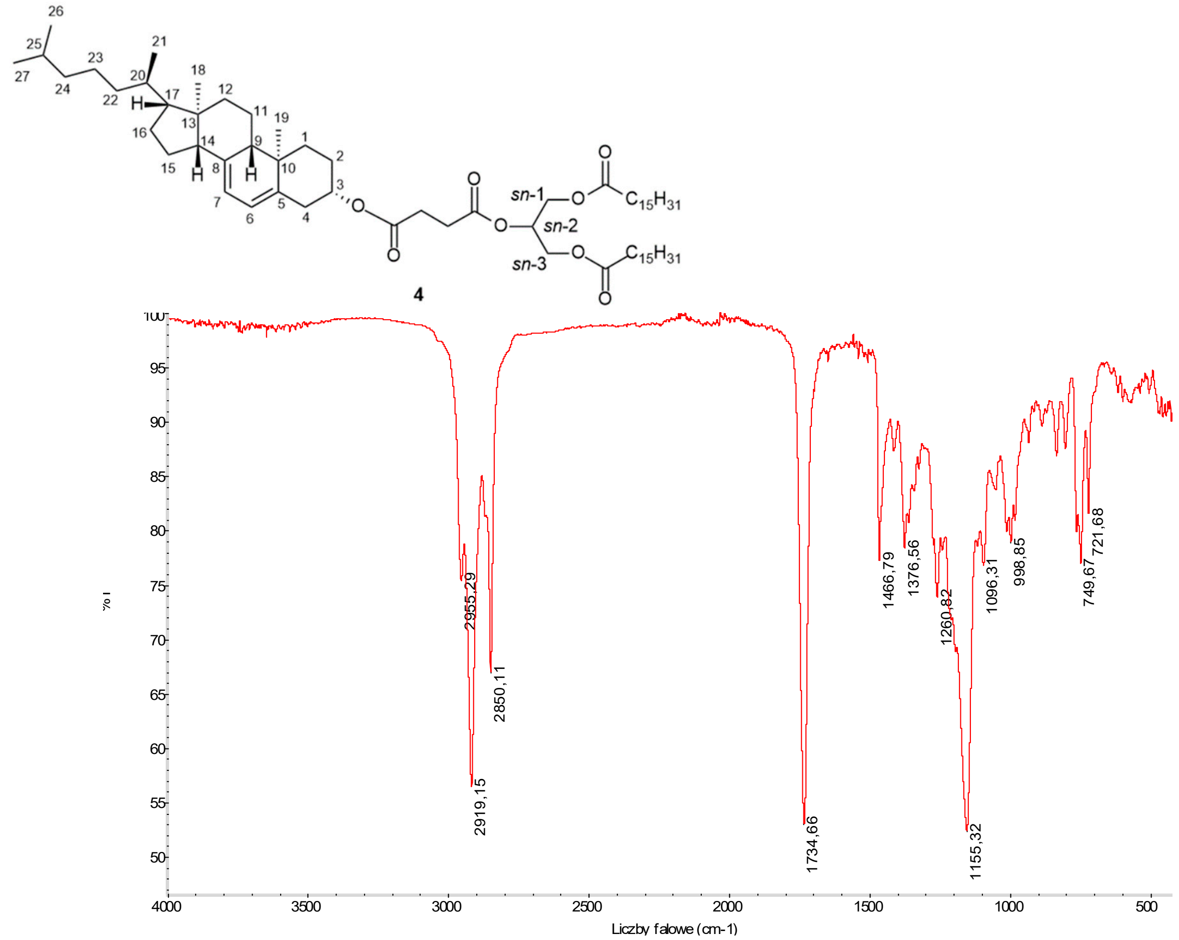

Figure S31. IR spectrum of 1,3-dipalmitoyl-2-(7-dehydrocholestyrylsuccinoyl)glycerol (**4**)

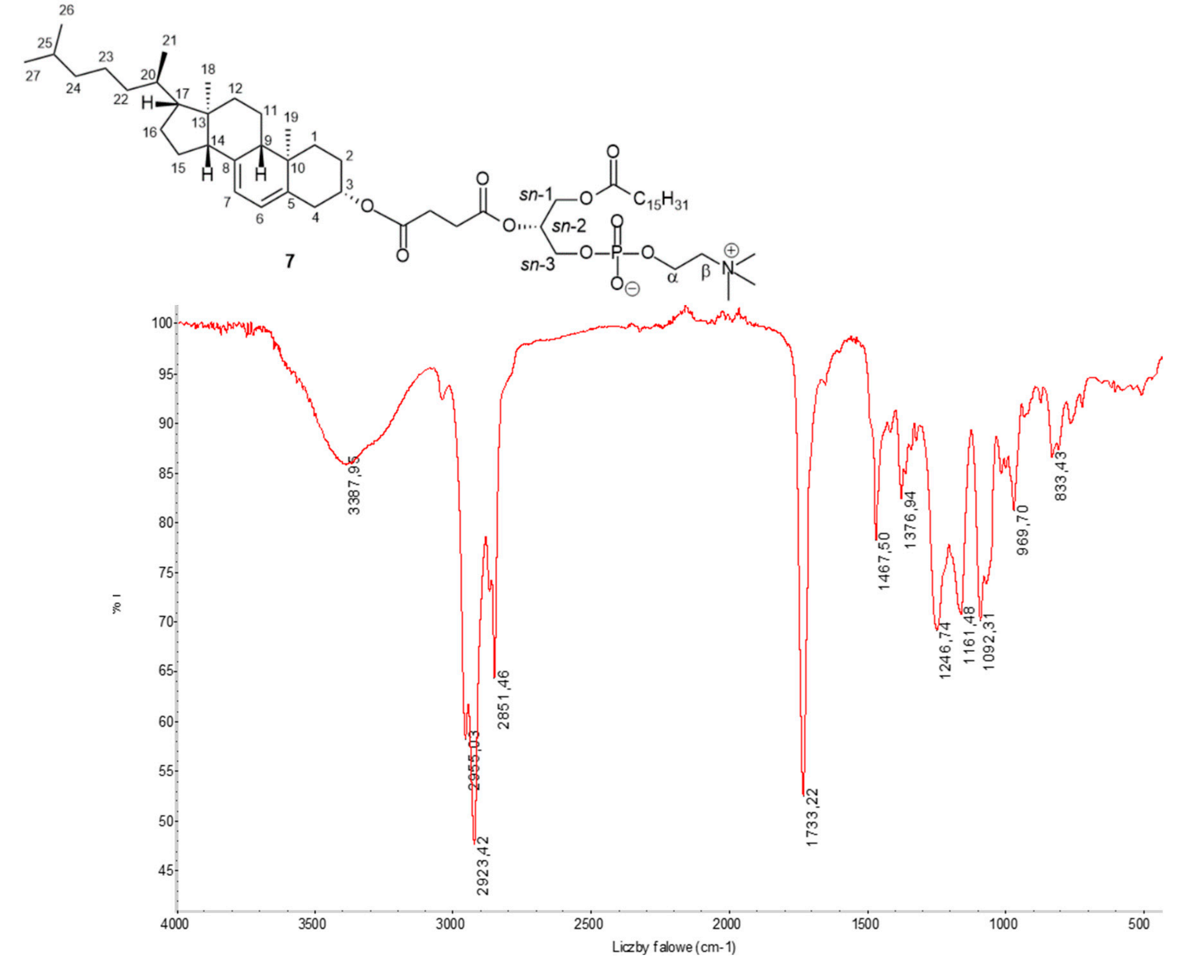

Figure S32. IR spectrum of 1-palmitoyl-2-(7-dehydrocholesterylsuccinoyl)-*sn*-glycero-3-phosphocholine (**7**)
